# Epoxides: Small Rings to Play with under Asymmetric Organocatalysis

**DOI:** 10.1021/acsorginorgau.2c00009

**Published:** 2022-03-29

**Authors:** Sara Meninno, Alessandra Lattanzi

**Affiliations:** Dipartimento di Chimica e Biologia “A. Zambelli”, Università di Salerno, Via Giovanni Paolo II, 84084 Fisciano, Italy

**Keywords:** asymmetric organocatalysis, epoxides, desymmetrization, kinetic resolution, Meinwald rearrangement, one-pot reaction

## Abstract

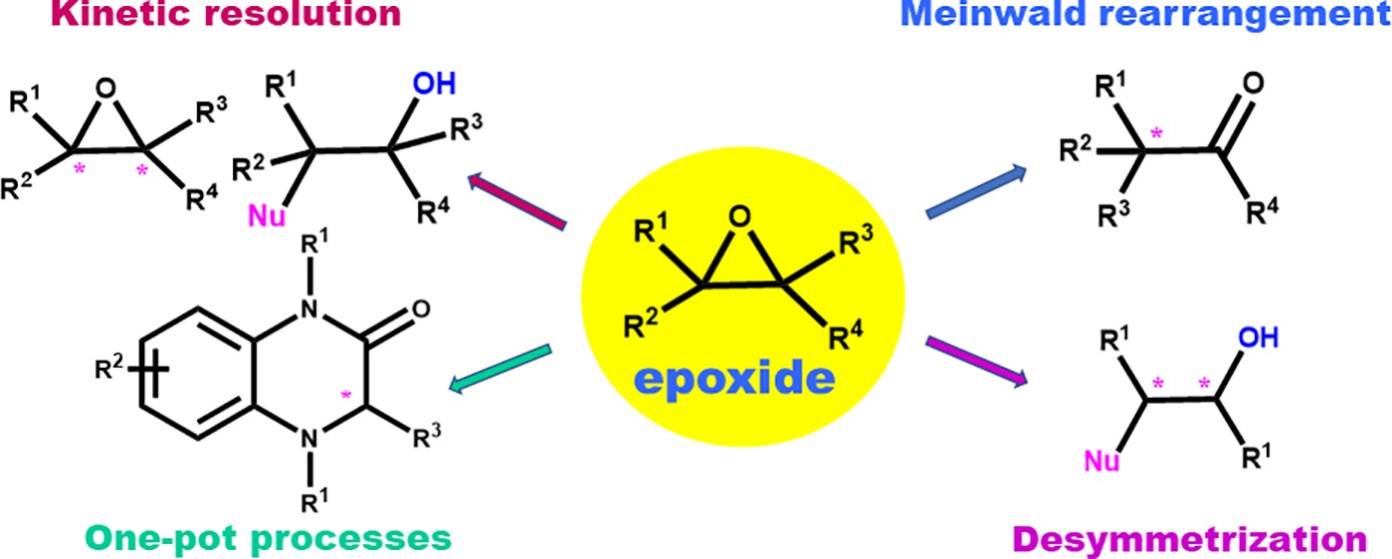

Optically pure epoxides are recognized as highly valuable products and key intermediates, useful in different areas from pharmaceutical and agrochemical industries to natural product synthesis and materials science. The predictable fate of the ring-opening process, in terms of stereoselectivity and often of regioselectivity, enables useful functional groups to be installed at vicinal carbon atoms in a desired manner. In this way, products of widespread utility either for synthetic applications or as final products can be obtained. The advent of asymmetric organocatalysis provided a new convenient tool, not only for their preparation but also for the elaboration of this class of heterocycles. In this review, we focus on recent developments of stereoselective organocatalytic ring-opening reactions of *meso*-epoxides, kinetic resolution of racemic epoxides, and Meinwald-type rearrangement. Examples of asymmetric organocatalytic processes toward specific synthetic targets, which include ring opening of an epoxide intermediate, are also illustrated.

## 1 Introduction

The synthesis of enantiomerically enriched molecules is of tremendous interest because their products are of pivotal importance in everyday life and in several industrial applications.^1−3^ The availability of one enantiomeric form of a compound is often a necessity in the pharmaceutical and agrochemical markets, due to the different physiological effect one enantiomer can show after interacting with the biological target, which is a chiral macromolecule.^4,5^

Among the tools available for their preparation, asymmetric organocatalysis is increasingly becoming a first choice, due to the mild and environmentally friendly working conditions, many activation strategies, and easy availability of the most common organocatalysts from natural sources.^6−8^

Optically enriched epoxides are undoubtedly the most versatile and useful heterocyclic compounds in organic synthesis, with several applications reported in natural and bioactive product preparation and in medicinal chemistry as intermediates or final drugs.^9−12^ Indeed, they behave as mild electrophilic compounds, whose significant inherent ring strain is released via S_N_2 displacement in the opening reaction, which can be accomplished under different catalytic conditions. Hence, the stereochemical outcome can be controlled, although the regioselectivity issue of the process is somewhat substrate-dependent. Accordingly, using common heteroatom- and carbon-centered nucleophiles, a countless number of valuable enantioenriched 1,2-difunctionalized products have been prepared, including, among others, amino alcohols, diols, halohydrins, cyanohydrins, hydroxysulfides, and alcohols.^13−16^

In the last two decades, many investigations focused on the asymmetric organocatalytic epoxidation of alkenes as the most straightforward approach to optically active epoxides,^17−22^ which in turn serve as the starting material for ring-opening reactions under achiral catalytic conditions in the presence or absence of a nucleophile to give the desired optically active final products.

**Figure 1:**
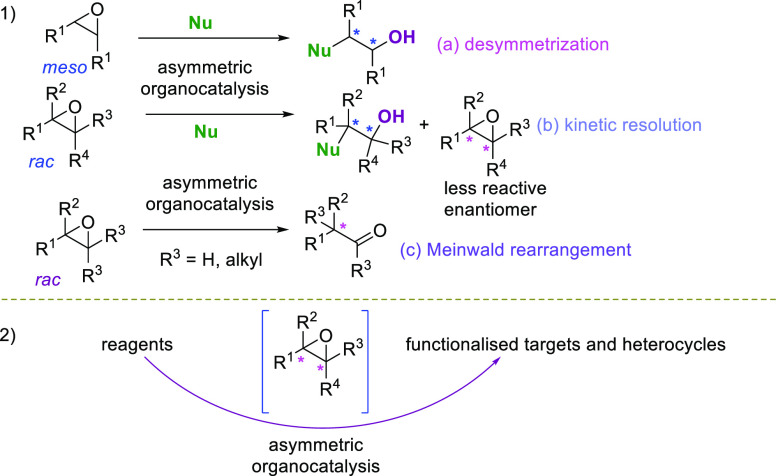
Ring-opening reactions to functionalized optically active molecules and heterocycles via *meso*- and racemic epoxides under asymmetric organocatalysis (1), via an asymmetric organocatalytic route involving a key epoxide intermediate (2).

The other way to access enantioenriched functionalized products makes use of *meso*- and racemic oxiranes in the ring opening or rearrangement events (Figure 1(1)).^15,23−119^ Due to the chiral environment established by the reagents and the optically pure organocatalyst, one of the two enantiotopic carbon atoms of the *meso*-epoxide selectively undergoes a S_N_2 nucleophilic displacement (Figure 1(1a)). This symmetry breaking transformation represents a powerful strategy of high synthetic value to install more chiral centers concurrently, taking into account that the starting epoxides are readily available reagents.

Similarly, another useful and versatile approach relies on the kinetic resolution of racemic epoxides with different nucleophiles (Figure 1(1b)).^25−27^ In an ideal case, two satisfactorily enantioenriched products can be obtained in 50% maximum yield when the ring-opening process displays perfect regioselectivity and the two enantiomers react with the nucleophile at significantly different rates (*S* = *k*_fast_/*k*_slow_ > 30).

It is worth noting that Lewis or Brønsted acid catalyzed Meinwald rearrangement is another useful process to obtain optically enriched carbonyl compounds.^28−31^ The migrating group generally attacks in an antiperiplanar direction to the C–O bond of the epoxide,^32^ thus assuring a predictable chirality transfer from the starting epoxide to the α-position of the carbonyl compound.

However, the control of regioselectivity is affected by the substitution pattern of the epoxide and the catalytic system used. Hence, a mixture of carbonyl compounds is generally observed. Although the migrating attitude can be difficult to control, simplification of the Meinwald rearrangement has been achieved using racemic terminal or tetrasubstituted epoxides, bearing two identical groups at one of the ring carbon atoms. A catalyst-controlled desymmetrization via hydrogen or alkyl shifts, allows the preparation of valuable enantioenriched aldehydes or challenging ketones, bearing α-all-carbon quaternary stereocenters (Figure 1(1c)).

The availability of simple and effective organocatalytic procedures for the synthesis of optically enriched epoxides facilitated the application of ring-opening reactions to access useful functionalized targets and heterocyclic compounds either under stepwise or one-pot conditions (Figure 1(2)). Different organocatalysts proved to be effective in the above-mentioned processes, mostly chiral phosphoric acids and to a lesser extent chiral Brønsted bases, N-heterocyclic carbenes, H-bonding donors, and polysaccharides.

Several reviews illustrated the ring opening of epoxides of strong chiral lithium bases and their applications in drug and bioactive product synthesis.^33−36^ However, the aim of this review is to focus the attention on recent organocatalytic asymmetric ring-opening reaction (ARO) and rearrangement of *meso*- and racemic epoxides, which have been reported since 2016, to improve the advances with respect to a previous survey on this topic.^37^ Moreover, examples including the elaboration of enantioenriched epoxides are illustrated to showcase the opportunities offered by the organocatalytic tool in target-specific asymmetric synthesis. The review is sectioned according to the ring-opening processes illustrated in Figure 1, which appear in the literature from 2016 to the end of 2021.

## 2 Desymmetrization of *meso*-Epoxides

In previous decades, the chiral ligand metal-based systems exemplified by Cr- and Co-salen^38,39^ Zn-trialkanol amine,^40^ Ti-^41^ and LiGa-BINOL,^42^ and Sc-bipyridine^43^ have been among the most extensively applied for the desymmetrization of *meso*-epoxides with heteroatom-centered nucleophiles. More challenging ring openings have seen the use of chiral Lewis bases/SiCl_4_ systems,^44^ including those involving carbon-centered nucleophiles^13,45−48^ and stoichiometric chiral lithium bases that were used to obtain functionalized allylic alcohols.^34,35,49^

In the realm of organocatalysis, chiral BINOL-, SPINOL-, and VAPOL-derived phosphoric acids have been the most extensively employed in organocatalysis, offering an excellent level of stereocontrol.^50−53^ Arguably, they can be considered among the most useful and versatile organic catalysts. Being bifunctional compounds, they are able to completely transfer protons or act as H-bonding donors, according to the nature of the reagents involved, thus demonstrating a wide degree of application in mechanistically different processes. Clearly, the ARO of epoxides has been one of the suitable reactions, amenable for application. The successful ARO of *meso*-epoxides, promoted by chiral phosphoric acids, was initially reported by the groups of Sun^54^ and List^55^ with sulfur- and carboxylic-acid-based nucleophiles, and this knowledge further inspired developments of the process.

**Scheme 1:**
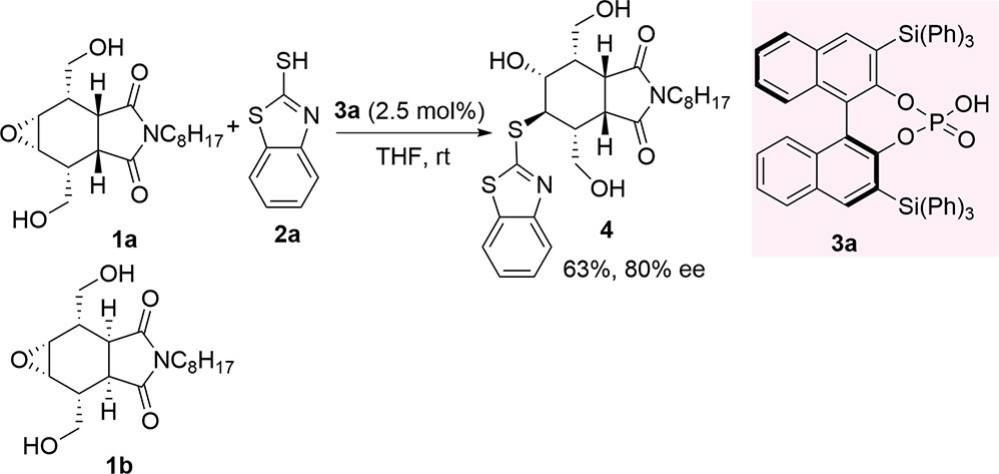
ARO of a *meso*-Diol Epoxide with 2-Mercaptobenzothiazole Catalyzed by BINOL-Derived Phosphoric Acid **3a**

In 2018, Johnson et al., with a view to obtain densely functionalized cyclohexane scaffolds, reacted diol epoxide **1a** with 2-mercaptothiazole **2a** in the presence of 2.5 mol % of BINOL-derived phosphoric acid **3a** in THF at room temperature (Scheme 1).^56^

Interestingly, the triol derivative **4**, bearing six contiguous chiral centers, was obtained in 63% yield and 80% ee in a single operation. Unfortunately, a less effective desymmetrization process was observed when using the *meso*-diastereoisomer **1b**, readily obtainable from **1a** under basic epimerization.

More recently, new chiral Brønsted acids have been synthesized and employed in the desymmetrization of *meso*-epoxides. In 2016, Lambert and co-authors first synthesized an enantiopure pentacarboxycyclopentadiene (PCCP)-based strong Brønsted acid via transesterification of easily available 1,2,3,4,5-pentacarbomethoxycyclopentadiene platform and (−)-menthol.^57^ This modular class of organocatalysts, in contrast with phosphoric acids, benefits from a short synthesis and a rich number of optically pure alcohols available to rapidly create libraries of acids, useful at the optimization stage of the asymmetric processes.

**Scheme 2:**
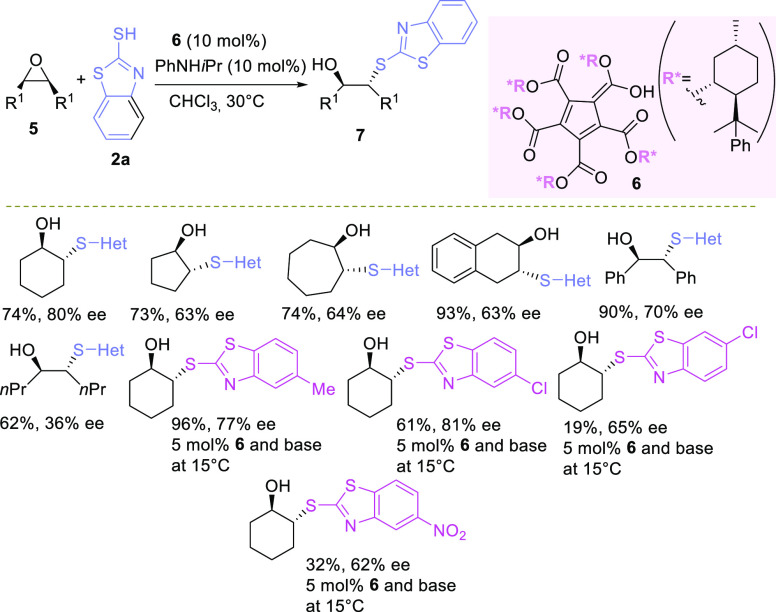
ARO of *meso*-Epoxides with 2-Mercaptobenzothiazoles Catalyzed by PCCP-Based Chiral Acid **6**

In 2018, Li’s group prepared (−)-8-phenylmenthol PCCP **6** and applied it in the desymmetrization of *meso*-epoxides with 2-mercaptothiazoles **2** (Scheme 2).^58^ Optimized reaction conditions required 10 mol % of catalyst **6** and an equivalent amount of *N*-isopropylaniline as an additive in chloroform at 30 °C. The presence of aniline and other aromatic bases as an additive proved to be helpful to control the enantioselectivity. The basic additive has been thought to be associated with either hydrogen bonding to form adducts or ammonium salts with the PCCP-based acid catalyst or π–π stacking interactions.

A variety of cyclic epoxides and stilbene oxides were converted to the desired alcohol derivatives **7** in good yields and up to 80% ee. The reaction appeared less successful when an acyclic aliphatic epoxide was used. A significant effect of the substitution pattern in the nucleophile **2** was observed with cyclohexene epoxide ring opening, especially with electron-withdrawing groups, likely ascribed to solubility and nucleophilicity issues. Scale-up to 5 mmol of cyclohexene oxide reaction with **2a** and recyclability of catalyst **6** for other runs was also demonstrated.

**Scheme 3:**
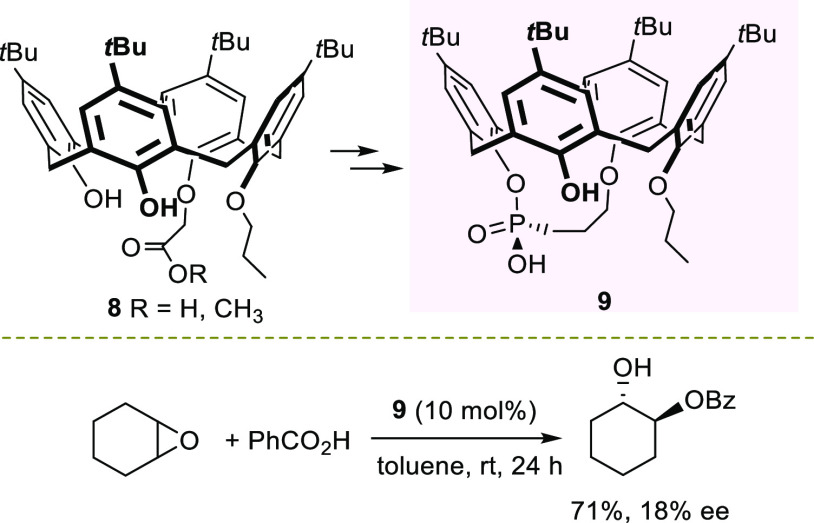
ARO of Cyclohexene Oxide with Benzoic Acid Catalyzed by Chiral Calixarene-Based Phosphonic Acid **9**

The well-known ability of calixarenes to give rise to host–guest complexation has fostered their application in chiral recognition and asymmetric catalysis.^59−61^ In 2018, Manoury et al. reported the synthesis of the first enantiopure inherently chiral calixarene-based phosphonic acid **9**.^62^ This catalyst was obtained via a four-step sequence from precursor acetic acid **8** or its methyl ester in 66% overall yield (Scheme 3).

The authors reported only a few examples of ARO reactions using exclusively benzoic acid as the nucleophile. The reaction performed on cyclohexene oxide catalyzed by 10 mol % of catalyst **9** proceeded smoothly, although the product was obtained with only 18% ee.

One of the most useful desymmetrization processes involves aminolysis of epoxides, given the widely recognized value of optically enriched 1,2-amino alcohol products as drugs, intermediates, or ligands for metal catalysis.^63−66^

**Scheme 4:**
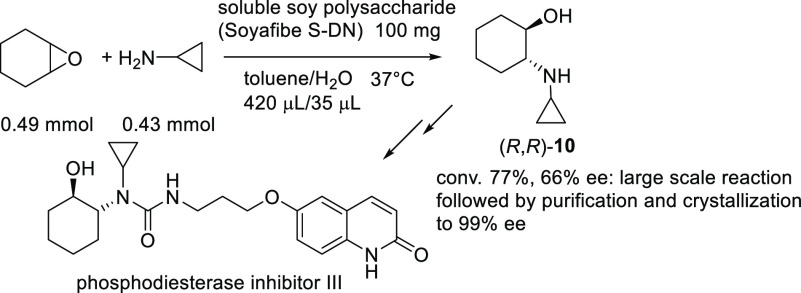
Asymmetric Amination of Cyclohexene Oxide with Cyclopropyl Amine Catalyzed by Soy Polysaccharide

In 2018, Takeuki and co-authors illustrated a target-oriented desymmetrization of cyclohexene oxide with cyclopropyl amine (Scheme 4).^67^ Optically pure (*R*,*R*)-amino alcohol **10** is the reagent for the synthesis of a phosphodiesterase III inhibitor, aging against vascular hypertrophy. The authors disclosed that the real catalyst was a polysaccharide contained in commercial soy bean flour, namely, the food additive Soyafibe S-DN, used as the catalyst.

A mixture of toluene and water assured a good conversion to product (*R*,*R*)-**10** obtained with 66% ee. An optimized process of purification and crystallization, also scalable at multigram scale (35 g of cyclohexene epoxide), enabled the amino alcohol to be obtained in 99% ee. The desymmetrization of cyclohexene oxide was performed with other primary amines, achieving modest results in terms of enantioselectivity (18–81% ee), whereas the epoxide scope appears limited to six- and five-membered epoxides.^68^ The presence of water was found to be necessary for the catalytic activity, likely controlled by the water-modified chain of the polysaccharide.

In 2020, Vicario et al. illustrated an innovative approach for the asymmetric synthesis of tropanes, based on intramolecular desymmetrization of *meso*-epoxides.^69^

**Scheme 5:**
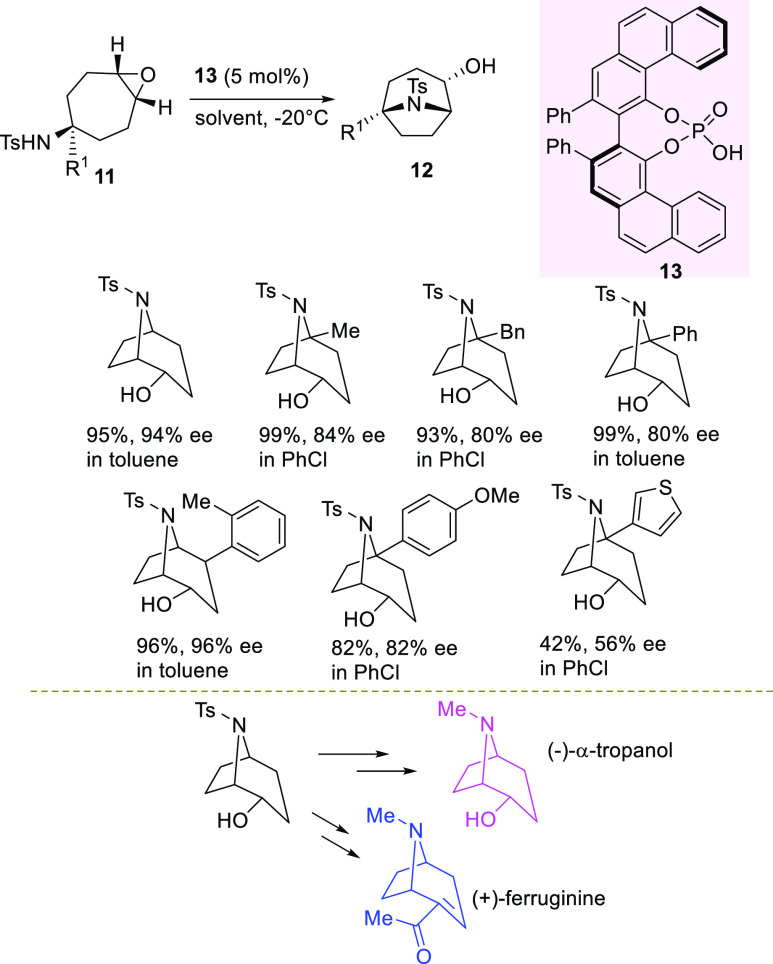
VAPOL-Based Phosphoric-Acid-Catalyzed Desymmetrization of 1-Aminocyclohept-4-ene-Derived Epoxides to Tropanols

The central scaffold of tropane alkaloids, namely, the 8-azabicyclo[3.2.1]octane core, has been recurrently targeted^70^ since the pivotal synthesis of tropinone reported by Robinson in 1917.^71^*meso*-4,5-Epoxycycloheptylamines **11** were screened with different chiral phosphoric acids, and finally VAPOL-derived organocatalyst **13** proved to be the most effective at 5 mol % loading in either toluene or chlorobenzene as the solvents at −20 °C. The presence of the electron-withdrawing tosyl group at nitrogen on the reactive 1,5-*trans*-diastereoisomer **11** was found to be crucial for the reaction to proceed. A variety of tropanols **12**, bearing hydrogen, alkyl, aryl, or heteroaryl substituents at the C1 carbon atom, were isolated in high yields and good to high ee values. Density functional theory (DFT) calculations confirmed a S_N_2 displacement in the ring-opening process, where the chiral acid promoted the hydrogen atom transfer from nitrogen to oxygen. Elaboration of products **12** for the asymmetric preparation of bioactive (−)-α-tropanol and the potent neurotoxine (+)-ferruginine was also successfully demonstrated (Scheme 5).

**Scheme 6:**
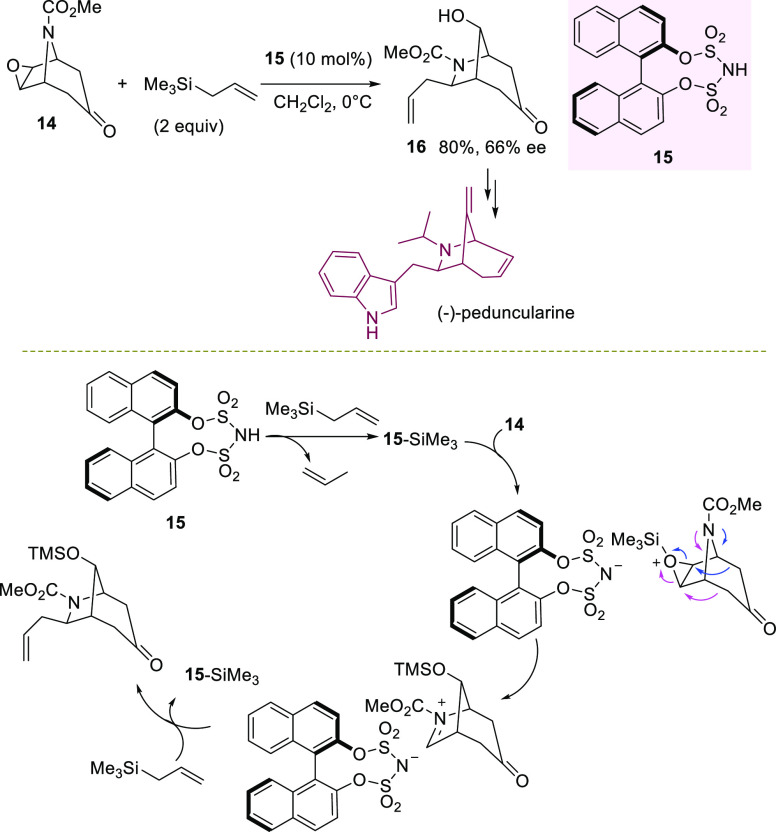
BINOL-Derived Bis(sulfuryl)imide-Catalyzed Desymmetrization of Epoxytropinone

In the same context, Hogson et al. accomplished the desymmetrization of the epoxytropinone **14** in the presence of allyl trimethylsilane to access a crucial intermediate **16**, which is useful for the asymmetric synthesis of (−)-peduncularine alkaloid (Scheme 6).^72^

After investigation with different catalytic systems, acidic BINOL-derived bis(sulfuryl)imide **15** performed at best in dichloromethane at 0 °C, affording intermediate **16** in 80% yield and 66% ee. This is a peculiar example of desymmetrization, where a skeletal rearrangement occurs in combination with carbon–carbon bond formation from an external nucleophile. Mechanistically, the organocatalyst underwent silylation by allyl trimethylsilane, giving the real catalytically active species **15**-SiMe_3_, which is able to silylate the epoxide, leading to an oxonium ion. The latter is engaged in a chiral environment with the catalyst anion, and it can selectively undergo ring opening with σ-bond participation to give an enantioenriched iminium intermediate. This species ultimately is captured by the external nucleophile, affording the product and restoring the **15**-SiMe_3_ species. It is likely to expect an improvement of the process to prepare intermediate **16** using modified BINOL-derived bis(sulfuryl)imides.

## 3 Kinetic Resolution

The most striking examples for the kinetic resolution of a broad variety of racemic terminal epoxides with different nucleophiles have been reported by the group of Jacobsen under Cr- and Co-salen catalysis.^9,38,39^ The perfect regioselectivity and the stereoselectivity factors, often comparable to those observed in the hydrolase-mediated resolutions,^73^ provided a versatile tool for the asymmetric synthesis of functionalized alcohols and terminal epoxides. Although the maximum 50% yield of both products is achievable, this process also offers a solution to the difficult task of preparing optically active terminal epoxides. Over the years, this research area has experienced further improvements in terms of heterogeneous and recyclable versions of the metal catalysts that can be employed, thus achieving an excellent level of practicality and applications at an industrial scale.^74^

Concerning the organocatalytic approach, amino thioureas served as the first promoters in the aminolytic kinetic resolution of racemic nitroepoxides,^75^ but more recently, Brønsted acid and H-bonding promoters appeared on the stage.

In 2016, List et al.^76^ illustrated an original catalytic approach for the asymmetric synthesis of uncommon heterocyclic compounds such as thiiranes, useful for constructing sulfur-containing derivatives and as chemical probes for biological systems.^77,78^ Indeed, established methods relied on chiral reagents or auxiliares using thionating reagents.^79,80^

**Scheme 7:**
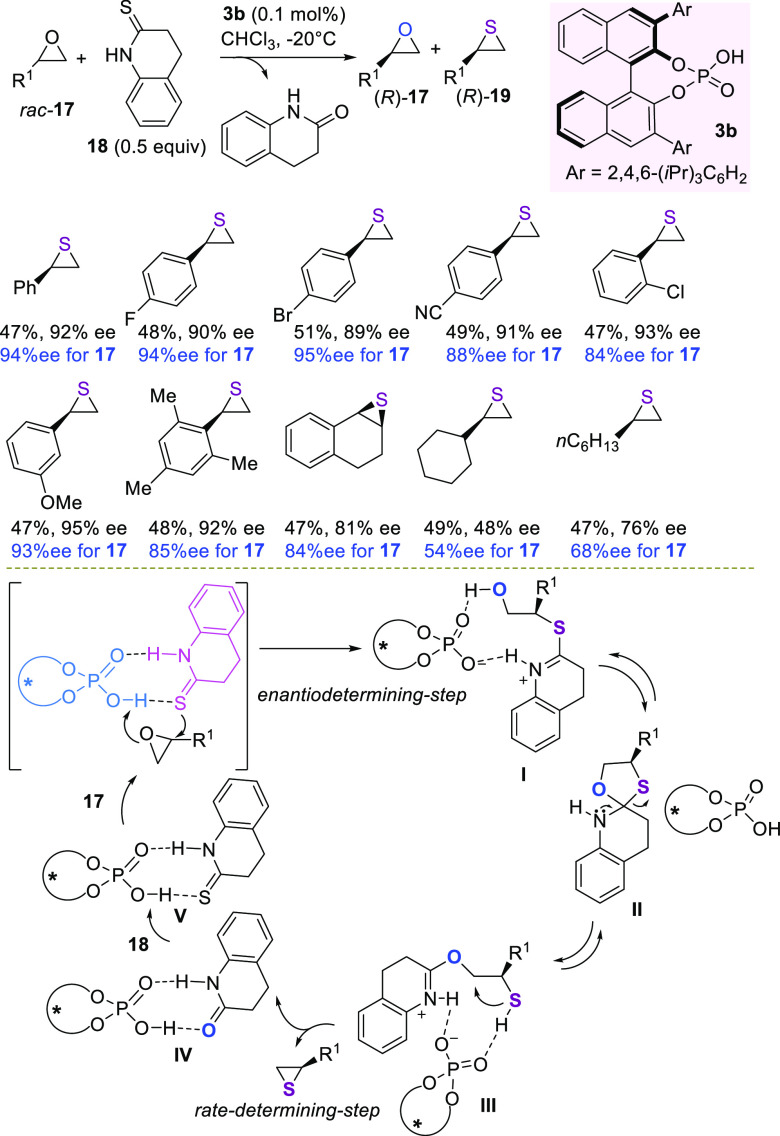
TRIP-Catalyzed Kinetic Resolution of Terminal Epoxides to Thiiranes

In previous work, the same group proposed a new activation strategy based on the formation of a heterodimeric H-bonding complex between the phosphoric acid and a carboxylic acid, necessary to activate the last one toward nucleophilic attack to *meso*-epoxides and to prevent alkylation of the organocatalyst by the alcohol formed in the ring-opening process.^55^ The noncovalent interactions in the self-assembled heterodimeric complex provide increased acidity of the phosphoric acid catalyst and increased nucleophilicity of the carboxylic acid. The same strategy has been successfully applied in the kinetic resolution of racemic terminal epoxides **17**, using thioamide **18** and well-known TRIP **3b** catalyst, at remarkably as low as 0.1 mol % loading (Scheme 7).

The kinetic resolution showed a wide substrate scope of aryl-substituted terminal epoxides, bearing electron-withdrawing or electron-donating groups at different positions of the phenyl ring. The corresponding thiiranes **19** were recovered in nearly 50% yield and excellent ee values, as well as the unreactive terminal epoxides. The stereoselectivity factors proved to be above 60, confirming a highly effective kinetic resolution process. Alkyl thiiranes were demonstrated to be more challenging substrates, being obtained with significantly lower ee values. The S_N_2 displacement was confirmed by the analysis on compounds **17** and **19**, both recovered with the *R*-absolute configuration. NMR investigations helped to formulate a mechanistic cycle involving a first attack by the sulfur reagent in the complexed heterodimer of one epoxide enantiomer to give intermediate **I**. This step was assumed to be enantioselectivity-determining. The latter would then cyclize to a 1:1 mixture of diastereoisomeric spirocyclic intermediate **II**, whose ring opening to intermediate **III** followed by the rate-determining ring closure to thiirane would complete the cycle. The lactam–TRIP heterodimer **IV**, concurrently formed, would then equilibrate with the reactive heterodimer **V** to initiate a novel catalytic cycle.

More recently, a DFT study of this reaction demonstrated the importance of sterically hindered *ortho*-substituents at the 3,3′-position of the aryl groups, as well as large *para*-substituents of the phosphoric acid catalyst, to improve the control of the enantioselectivity.^81^

**Scheme 8:**
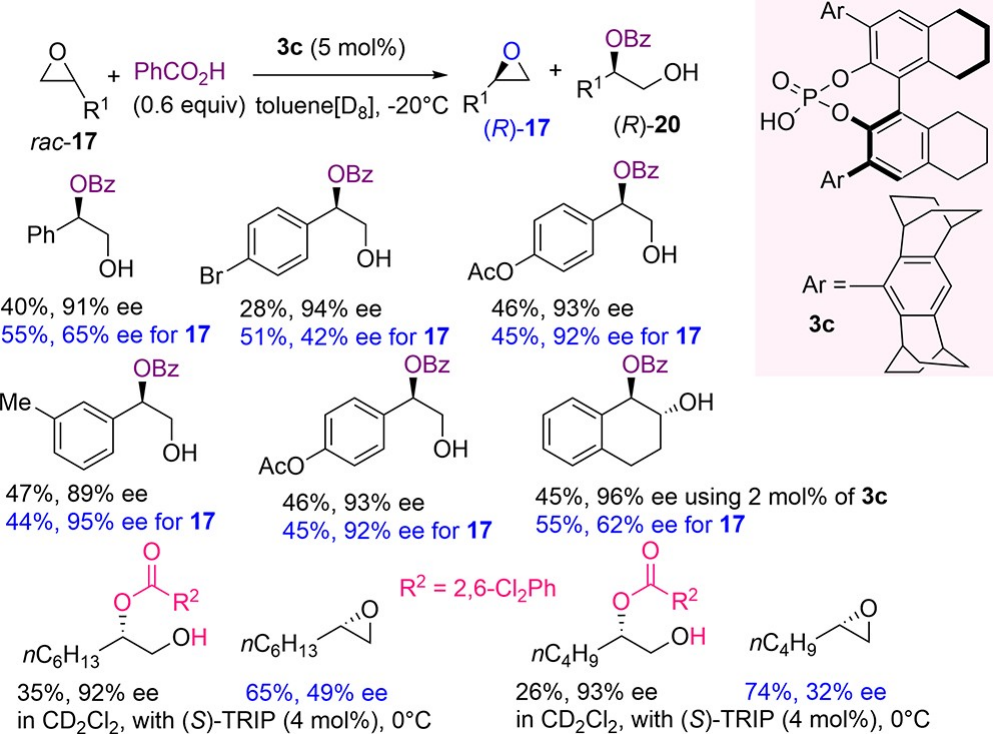
H_8_-BINOL-Derived Phoshoric-Acid-Catalyzed Kinetic Resolution of Racemic Terminal Epoxides with Carboxylic Acids

In the same year, List and co-workers expanded the applicability of the self-assembled activation strategy of phosphoric acid with carboxylic acids in the kinetic resolution of racemic terminal epoxides (Scheme 8).^82^

Specifically, benzoic acid was employed as the nucleophile, and more sterically demanding confined H_8_-BINOL-derived phosphoric acid **3c** was used to promote the reaction. Notably, complete control of the regioselectivity was observed with the formation of the O-benzolylated aryl ethylene glycols **20** in high yields and ee values. High stereoselectivity factors were estimated for the substrates (*S* ranging from 29 up to 93). By further investigation, an asynchronous S_N_2 ring-opening process was ascertained. With aliphatic terminal epoxides, to maintain the regioselectivity previously observed, fine optimization of the reaction conditions was required. However, it was possible to regioselectively resolve linear terminal epoxides with a substituted benzoic acid, maintaining the same stereocontrol at the expense of lower final yield of the protected glycols. DFT calculations provided useful insights on the transition state of the reaction, which guided additional development of this process.

**Scheme 9:**
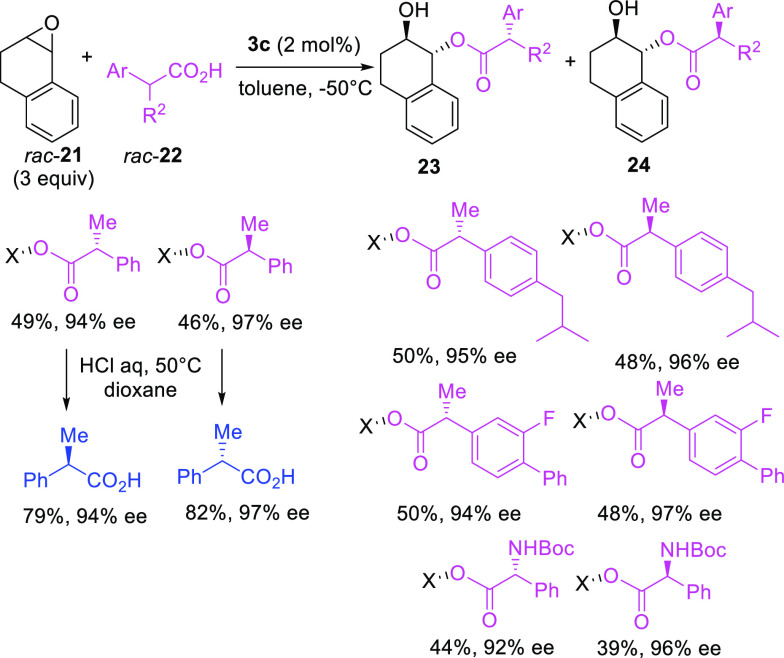
H_8_-BINOL-Derived Phoshoric-Acid-Catalyzed Kinetic Resolution of Racemic α-Chiral Carboxylic Acids with a Model Racemic Epoxide

Interestingly, the authors conceived an unprecedented stereodivergent parallel kinetic resolution of racemic α-chiral carboxylic acids (Scheme 9). The newly formed stereogenic centers in diastereoisomers **23** and **24** were fixed exclusively by the absolute configuration of the catalyst. Both enantiomers of racemic acid **22** ring-opened the most reactive enantiomer of the starting epoxide **21**, partitioning in equal amounts in diastereoisomers **23** and **24**, readily separable by chromatography. Under optimized conditions, medicinally relevant carboxylic acids and *N*-Boc-protected phenylglycine were resolved, achieving excellent levels of enantioselectivity, as demonstrated after hydrolysis of model diastereoisomers. After the kinetic resolutions, the authors recovered unreacted epoxide **21** enantioenriched from 58% up to 92% ee.

Spiro-epoxyoxindoles are privileged skeletons, endowed with several biological activities.^83,84^ In particular, terminal spiro-epoxyindoles have been scarcely investigated, and a handful of examples for their asymmetric preparation has been reported, although they are versatile intermediates for the synthesis of indole-based alkaloids.^85^

**Scheme 10:**
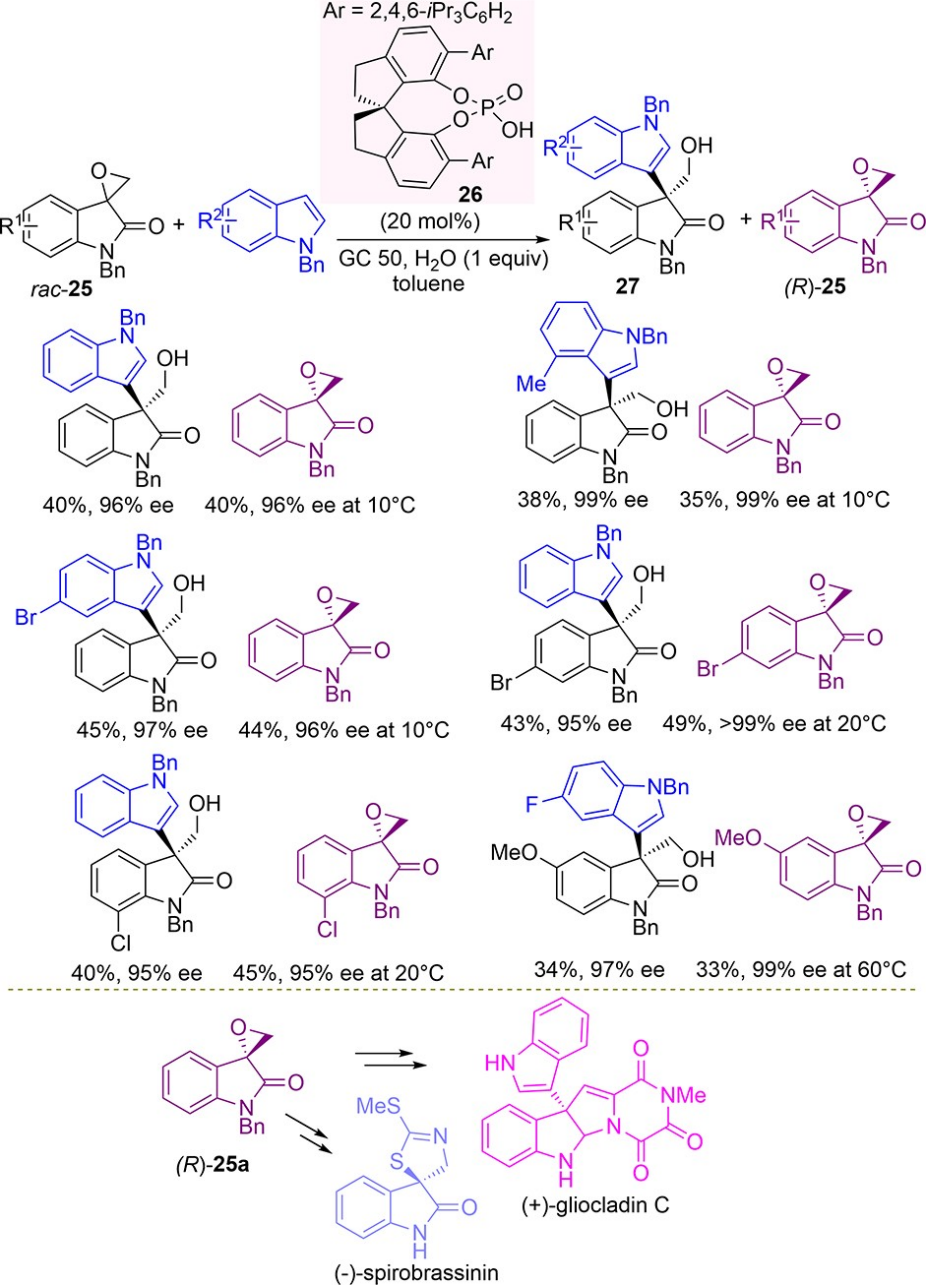
SPINOL-Derived Phoshoric-Acid-Catalyzed Kinetic Resolution of Racemic Terminal Spiro-Epoxyindoles in Friedel–Crafts Alkylation with Indoles

In 2017, Wang and co-authors envisioned a phosphoric-acid-catalyzed kinetic resolution of racemic terminal spiro-epoxyindoles **25** via Friedel–Crafts alkylation of indoles (Scheme 10).^86^

After fine optimization, SPINOL-based phosphoric acid **26** was found to be highly effective in the presence of weakly acidic Amberlite GC 50 and water as additives, working at different temperatures. A good variety of unreacted enantiomerically enriched (*R*)-epoxides **25** as well as alkylated products **27** were isolated, after prolonged reaction times, in high yields and excellent ee values, irrespective of the substitution pattern in the aromatic ring. Indeed, this process showed impressive stereoselectivity factors of up to 1060. The reaction was successfully scaled-up to 1 g of recovered (*R*)-**25a** with 98% ee, which was employed as the starting material for the formal enantioselective synthesis of fungal alkaloid (+)-gliocladin C and (−)-spirobrassinin, a natural product displaying antifungal^87^ and anticancer activities.^88^

One of the most recurrent applications of abundant C1 building block carbon dioxide (CO_2_) concerns its atom-economical incorporation into organic carbonates through reaction with epoxides.^89^ Only a few examples of kinetic resolution of racemic terminal epoxides with CO_2_ have been catalyzed by metal and organocatalytic systems.^90,91^

**Scheme 11:**
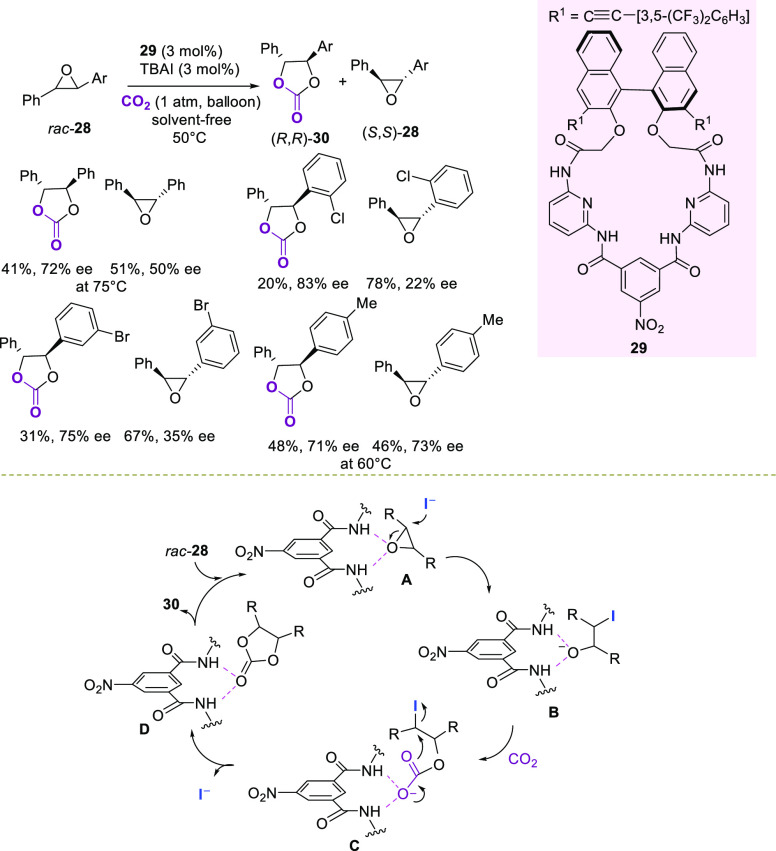
H-Bond-Catalyzed Kinetic Resolution of Racemic Disubstituted *trans*-Epoxides with CO_2_

Ema et al. recently illustrated a first example of kinetic resolution of disubstituted epoxides **28** with CO_2_ using a chiral macrocyclic catalyst **29**, acting as the H-bond donor (Scheme 11).^90^

Compounds of type **29** have been previously used in NMR chiral discrimination of different molecules, including epoxides, taking advantage of the amide NH groups.^91^ On the basis of these data, the authors hypothesized that promoter **29** would have been able to act as the H-bonding donor, activating one enantiomer of the epoxide toward CO_2_ insertion. The presence of catalytic loadings of tetrabutylammonium iodide (TBAI) was found to be necessary for the reaction to proceed. The kinetic resolution was simply performed under solvent-free conditions at 50 °C in the presence of 3 mol % of catalyst **29** and CO_2_ (1 atm). Cyclic carbonates **30** and the unreacted (*S*,*S*)-epoxides bearing electron-withdrawing and electron-donating groups in the phenyl ring were recovered in good yields and moderate enantioselectivity. Under the same reaction conditions, terminal racemic epoxides proved to be less efficiently resolved. With NMR analysis of epoxides and catalyst mixtures as a guide, a catalytic cycle has been proposed, where the (*R*,*R*)-enantiomer of **28** would be preferentially H-bonded with the amide NH groups in complex **A**.

Next, the iodide anion would attack the epoxide to give intermediate **B**, which undergoes CO_2_ addition to form an opened carbonate **C**. The latter would intramolecularly cyclize via S_N_2 displacement to provide the H-bonded carbonate **D**, which is then released in the reaction mixture. DFT calculations supported the H-bonding activation of the epoxide, occurring inside the macrocyclic cavity.

**Scheme 12:**
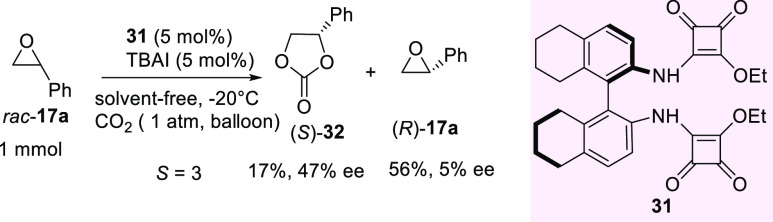
H_8_-Binaphthyl-Linked Hemisquaramide-Catalyzed Kinetic Resolution of Racemic Styrene Epoxide with CO_2_

In 2019, the same group disclosed a new class of H-bonding organocatalysts, such as H_8_-binaphthyl-linked hemisquaramides, able to accelerate the incorporation of CO_2_ into terminal epoxides to afford cyclic carbonates (Scheme 12).^92^ Similar to a previous investigation illustrated in Scheme 11, the reaction proceeded under solvent-free conditions using TBAI as the cocatalyst. Simple and readily available catalyst **31**, when used at 5 mol % loading, was found to be effective in the kinetic resolution of racemic styrene oxide **17a** at −20 °C (Scheme 12).

Under these conditions, carbonate **32** was recovered in 17% yield and 47% ee, whereas the unreacted epoxide was isolated as an almost racemic product. Given the low estimated selectivity factor (*S* = 3), further developments are needed to improve this approach as an effective tool to enantioenriched carbonates and epoxides. However, results illustrated in Schemes 11 and 12 highlight the importance of H-bonding catalysis as a viable and mild strategy to exploit for the kinetic resolution of epoxides other than chiral phosphoric acids.^93^

## 4 Meinwald Rearrangement

**Scheme 13:**
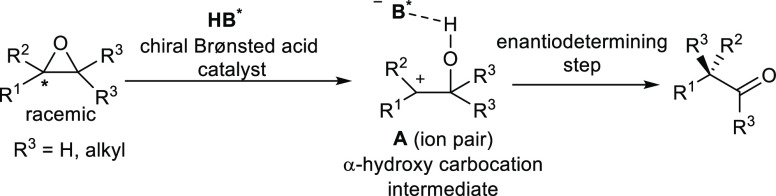
Mechanism of the Catalytic Enantioselective Meinwald Rearrangement

From a synthetic point of view, catalyst-controlled Meinwald rearrangements that are able to transform racemic epoxides into enantioenriched α-substituted carbonyl compounds are the most attractive approaches among semipinacol rearrangement reactions.^31^ In particular, using tetrasubstituted epoxides, it is possible to synthesize chiral ketones bearing α-all-carbon quaternary stereocenters. This approach serves as an effective alternative to the classical α-arylation or α-alkylation of ketone enolates, enabling some of the synthetic difficulties often met in the classical regioselective formation of a single enolate at different α-enolizable positions to be overcome. Moreover, the substrate scope is essentially limited to cyclic ketones, where the defined conformation of tetrasubstituted enolates makes it possible to control the stereochemistry of the reaction. However, despite its utility, the development of an enantioselective catalytic asymmetric Meinwald rearrangement is a challenging task. The chiral transfer between classic Lewis or Brønsted acid catalysts and the carbocation intermediate is not easy to achieve. Mechanistically, upon activation by acids, tetrasubstituted epoxides with only one stereogenic center undergo a regioselective ring-opening reaction, forming a prochiral α-hydroxy carbocation intermediate. When a chiral Brønsted acid is used as a catalyst, the enantioselective alkyl shift could potentially take place on the carbocation paired with the chiral counteranion of the deprotonated catalyst. The chiral ion pair interaction induces chirality, leading to enantioenriched ketones (Scheme 13).

**Scheme 14:**
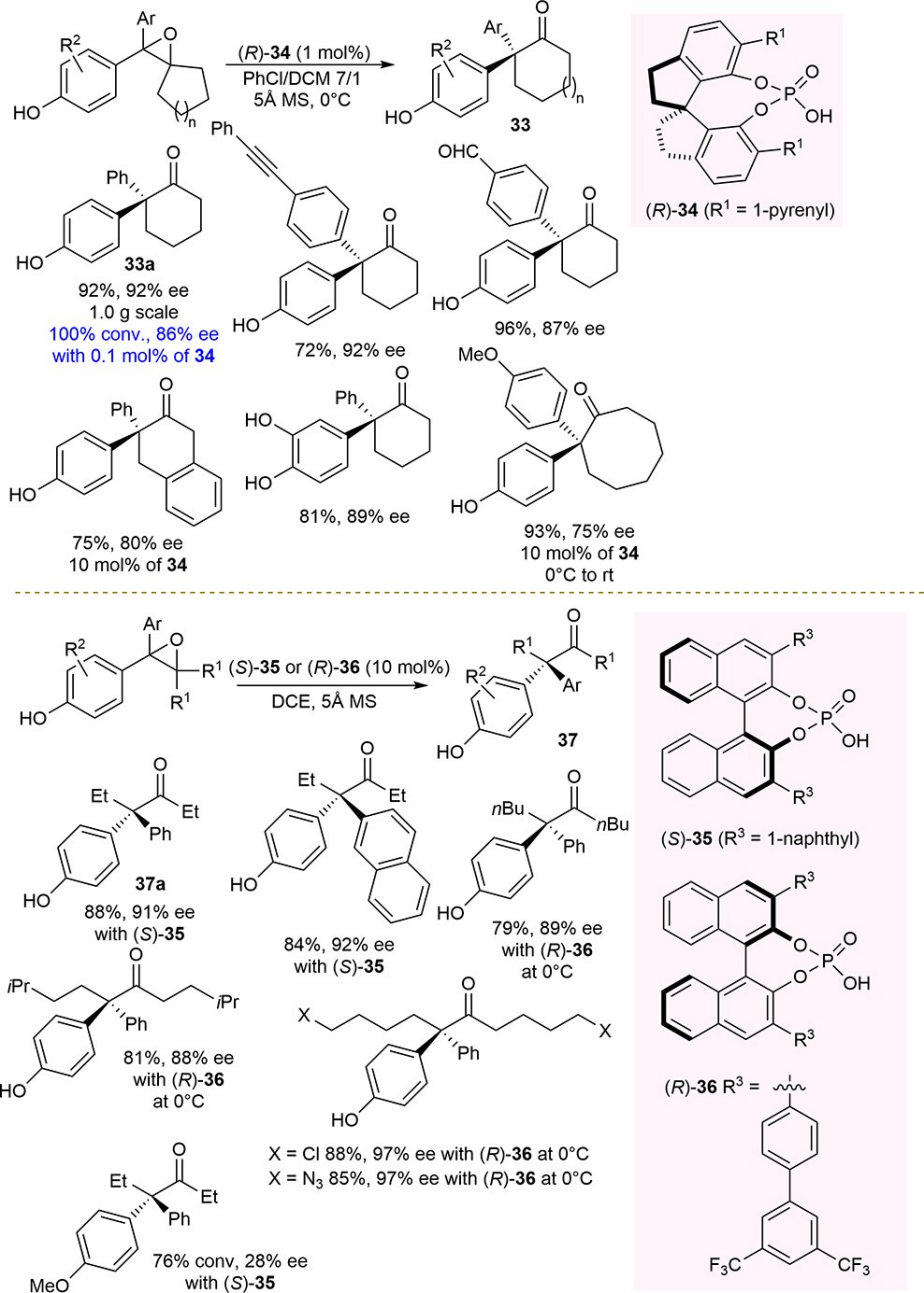
Catalytic Enantioselective Meinwald Rearrangement of Racemic Epoxides for the Synthesis of Ketones Bearing a Quaternary Stereocenter

This asymmetric stereoconvergent strategy was reported independently by Sun and Zhu in two elegant examples in 2019. Sun reported a chiral phosphoric-acid-catalyzed enantioconvergent Meinwald rearrangement of tetrasubstituted epoxides for the synthesis of both cyclic and acyclic ketones bearing α,α-diaryl quaternary stereocenters, inaccessible through classical methods (Scheme 14).^94^

The catalyst was found to be able to enantiodiscriminate two sterically similar aryl groups on the carbocation intermediate and, especially in the case of cyclic ketones, showed a high turnover. It could be used as low as 0.1 mol %, maintaining full conversion and a slightly lower ee value of the product. In the case of more challenging acyclic ketones, the protocol was effective also with epoxides bearing alkyl chains longer than those of methyl and ethyl groups, such as *n*-butyl groups as well as ones ending with chloride or azide functionalities.

**Scheme 15:**
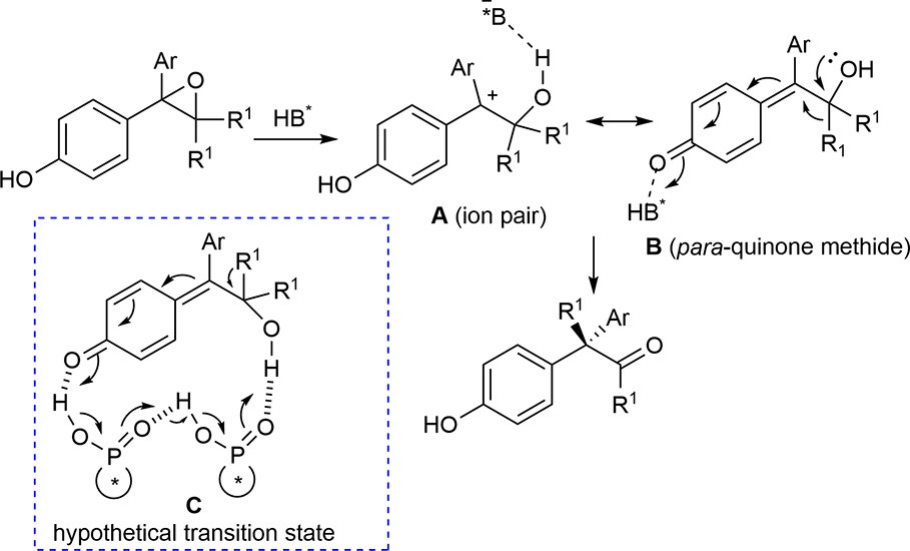
Proposed Mechanism of the Catalytic Enantioselective Meinwald Rearrangement of Racemic Epoxides

The presence of the *para*-hydroxy group was demonstrated to be necessary to reach a high level of enantioselectivity, thus validating the proposed mechanism which would involve the neutral *para*-quinone methide **B** as a pseudoresonance structure of the chiral ion pair (Scheme 15).

Positive nonlinear effects between the catalyst’s and product’s ee values, together with a kinetic order of 1.6 in the catalyst, suggested a transition state involving a higher-order catalyst aggregate **C** to be likely active. However, different catalytic species might be involved according to the structure of the epoxide. Finally, the synthetic utility of the ketone products was demonstrated in the preparation of a wide range of chiral molecules bearing all-carbon quaternary stereocenters.

**Scheme 16:**
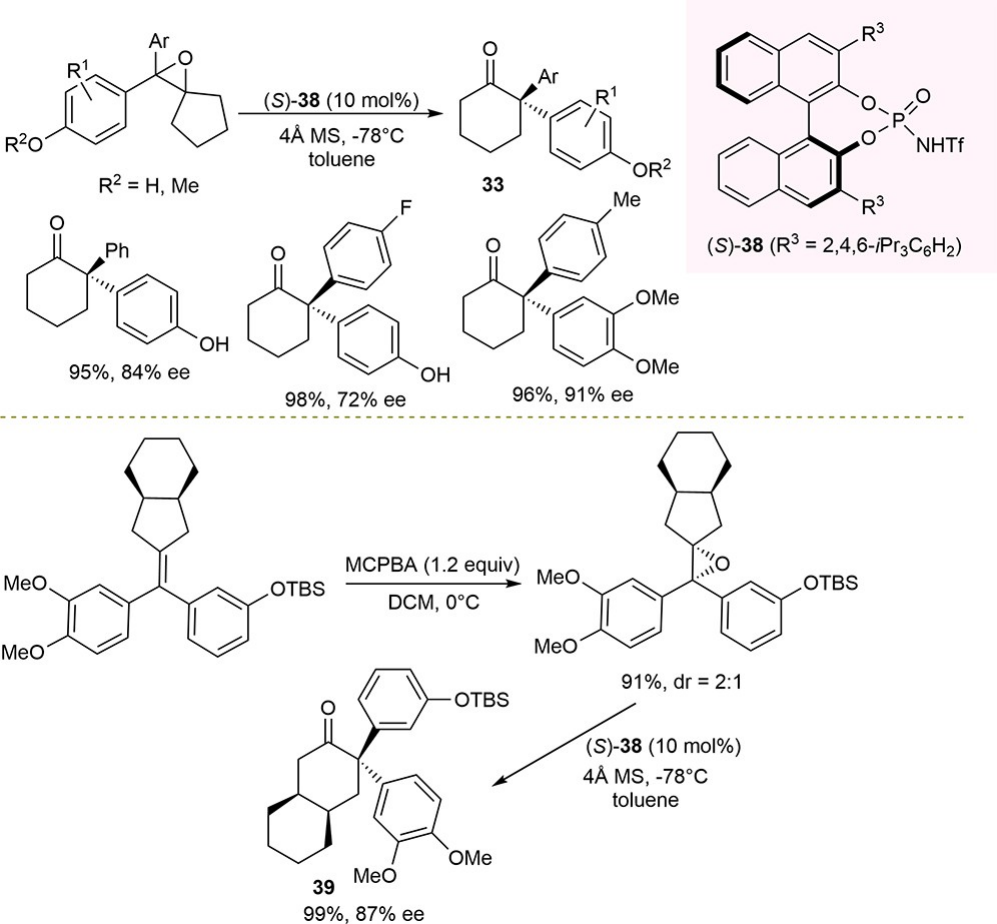
Catalytic Enantioselective Meinwald Rearrangement of Tetrasubstituted Racemic Epoxides and Desymmetrized Meinwald Rearrangement

Concurrently, Zhu described chiral *N*-triflyl phosphoramide-catalyzed enantioselective pinacol rearrangement of 1,2-tertiary diols and Meinwald rearrangement of tetrasubstituted epoxides for the synthesis of 2-alkynyl-2-arylcyclohexanones and 2,2-diarylcyclohexanones (Scheme 16).^95^

In addition, a desymmetrizing example of Meinwald rearrangement was reported, affording a difficult to access 3,3-diaryl-substituted bicyclic cyclohexanone **39**, bearing three stereocenters in 87% ee, broadening the substrate scope. Similarly to Sun’s work, the presence of molecular sieves was important for the reaction outcome. The data suggest that both H-bonding interactions, between the neutral quinone methide intermediate and the catalyst, and ion pairing interactions, between a carbenium intermediate and chiral phosphate, would be involved in the control of asymmetric induction.

## 5 Synthetic Processes Involving Epoxides as Intermediates

Epoxide-based intermediates are gaining attention in an increasing number of processes targeted to the stereoselective synthesis of heterocycles, drug candidates, and biologically active compounds. By exploiting their chemistry, dominated by regioselective ring-opening reactions with a wide range of nucleophiles, it has been possible to develop novel and, in many cases, more sustainable strategies for the asymmetric synthesis of otherwise difficult to access compounds, with a lot of applications in drug development.^96^ Moreover, recently, step-economic and sustainable methodologies recently succeeded in combining preparation steps of the epoxide from alkenes followed by regioselective ring opening in one pot and tandem organocatalyzed reactions, with enormous advantages from a green chemistry point of view.^97−99^

**Scheme 17:**
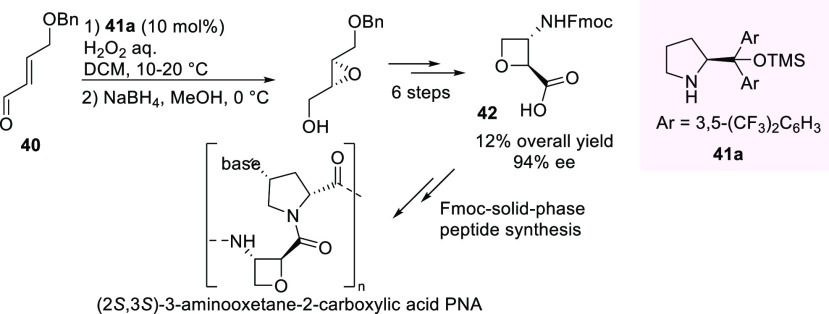
Synthesis of Optically Active Building Block (2*S*,3*S*)-*epi*-Oxetin via Enantioselective Epoxidation Promoted by Jørgensen Catalyst as the Key Step

In 2017, an enantioselective nucleophilic epoxidation of an aliphatic α,β-unsaturated aldehyde, (*E*)-4-benzyloxy)but-2-enal **40**, with aqueous hydrogen peroxide promoted by Jørgensen catalyst **41a**, was successfully exploited as a key step in a more concise synthesis of the building block, Fmoc-protected (2*S*,3*S*)-*epi*-oxetin **42** (Scheme 17).^100^ Intermediate **42** was obtained in 12% overall yield and 94% ee in an eight-step sequence, involving ring-opening reaction of epoxide with azide, activation of primary alcohol, ring closure, followed by simple transformations of functional groups. Fmoc-protected (2*S*,3*S*)-*epi*-oxetin **42** was used as a building block for the synthesis of a novel oxetane-containing pyrrolidinyl peptide nucleic acid (PNA) to study the effects of the oxetane-containing backbone in comparison to previously investigated conformationally constrained PNAs.

**Scheme 18:**
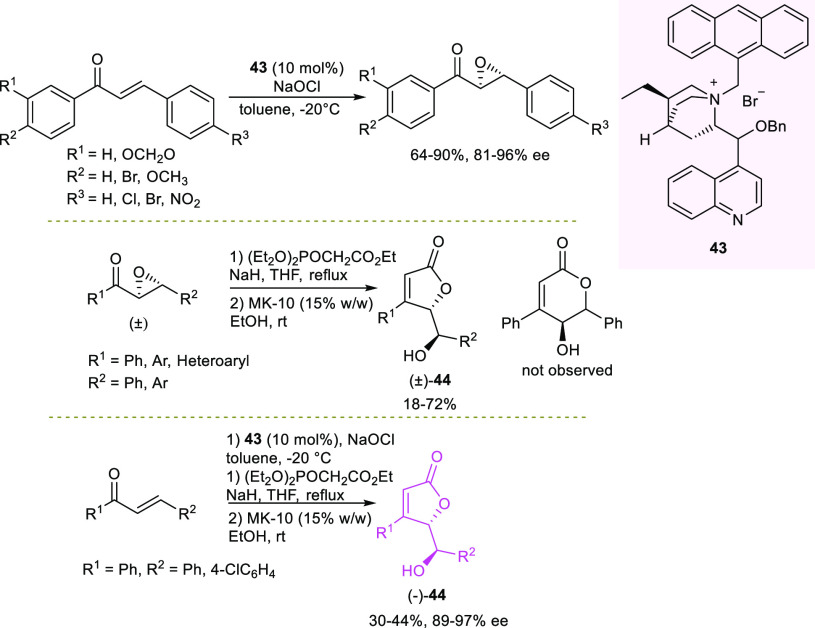
Three-Step Synthesis of Enantioenriched γ-Butenolides Exploiting Asymmetric Organocatalytic Epoxidation of Chalcones as the Key Step (MK-10: montmorillonite K10)

Concurrently, Corrêa and co-authors reported the stereoselective synthesis of hydroxybenzyl-substituted γ-butenolides **44**, starting from enantioenriched epoxyketones, obtained via organocatalytic epoxidation of chalcones with NaOCl and in the presence of *N*-antracenylmethyl-substituted cinchona-derived ammonium salt **43** (Scheme 18).^101^

The epoxidation step and the two-step one-pot sequence to obtain γ-butenolides from the corresponding epoxides were optimized separately. Epoxides were isolated from six differently substituted chalcones in 64–90% yield and 81–96% ee. The one-pot process, first optimized on racemic epoxides, involved Horner–Wadsworth–Emmons (HWE) olefination, followed by solvent replacement with greener ethanol to carry out the hydrolysis. Interestingly, the γ-butenolides were selectively formed in up to 72% yield, whereas the δ-pentenolides were not observed. The authors then demonstrated in two examples the synthesis of enantioenriched γ-butenolides through a three-step sequence, involving only one chromatographic purification of the final product. The γ-butenolides were recovered in moderate yield (30–44% yield) and high enantioselectivity (89–97% ee).

**Scheme 19:**
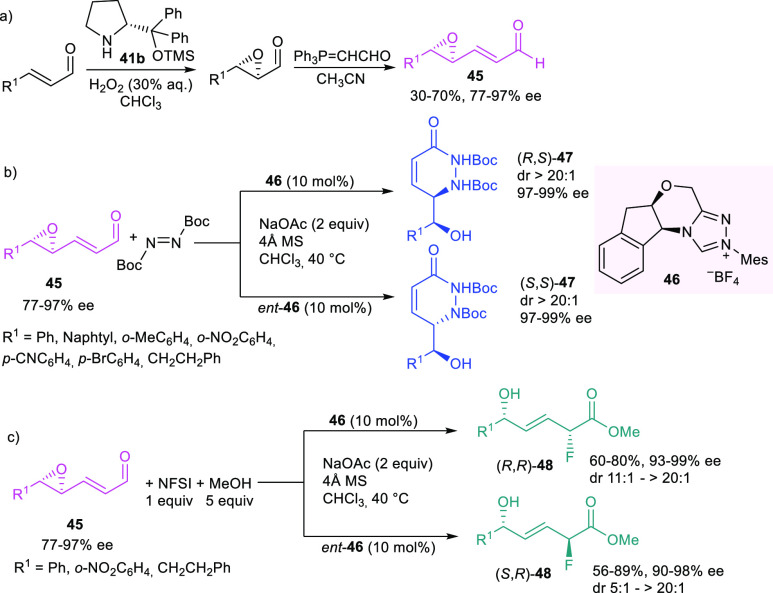
NHC-Catalyzed Regio- and Stereoselective Ring Opening of Epoxy Enals to Access 1,2-Amino Alcohols and 1,4-Fluoro Alcohols (NFSI: *N*-Fluorobenzenesulfonimide)

In 2018, Lu and Zhao reported a three-step procedure involving the enantioselective epoxidation via iminium catalysis/Wittig olefination/enantioselective vinylogous ring opening by N-heterocyclic carbene (NHC) catalysis to accomplish the regio- and stereodivergent synthesis of 1,2-amino alcohols and 1,4-fluoro alcohols (Scheme 19b,c).^102^

**Scheme 20:**
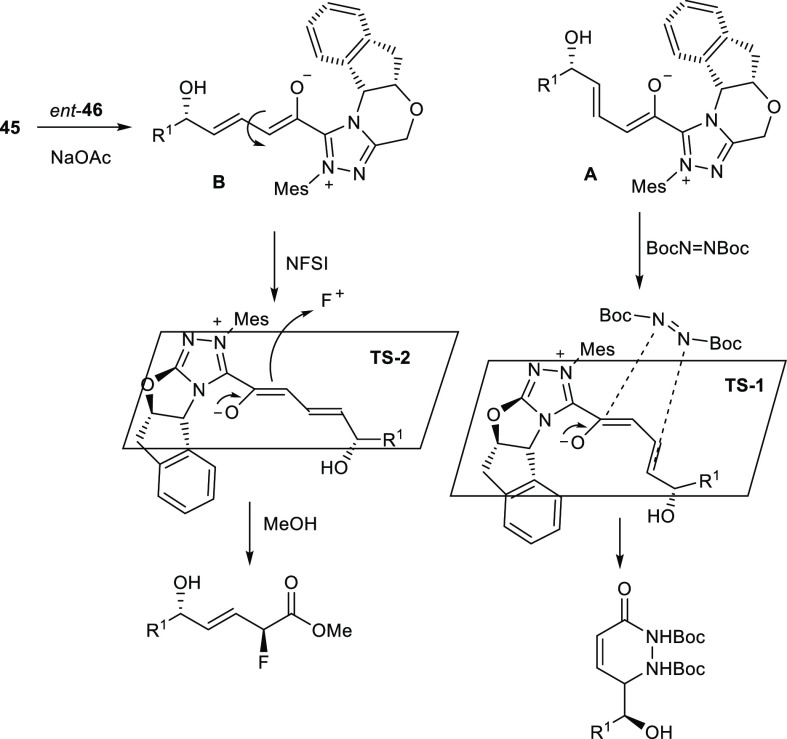
Hypothetical Transition States and Mechanism Involved in the Amination and Fluorination Reaction of Chiral Epoxy Enals

By choosing the proper enantiomer of the azolium catalyst **46**, in the presence of NaOAc as the basic additive, both (*R*,*S*) and (*S*,*S*)-amino alcohols **47** could be prepared with excellent diastereo- and enantioselectivity, starting from preformed highly enantioenriched epoxyenals **45**. Chiral epoxides **45** were easily prepared via OTMS-protected diphenyl prolinol organocatalyzed epoxidation of α,β-unsaturated aldehydes using H_2_O_2_ as the oxidant,^103,104^ followed by Witting olefination to install the strategically located vicinal enal group (Scheme 19a). The substrate scope of the stereodivergent NHC-catalyzed amination is quite general with respect to the nature of different aromatic and alkyl-substituted enals, affording either (*R*,*S*) or (*S*,*S*)-**47** with excellent diastereo- (>20:1 dr) and enantioselectivity (97–99% ee) (Scheme 19b). NHC catalysis proved to be highly effective in the control of distal stereocenters, promoting the enantioselective α-fluorination of epoxy enals **45** to access (*R*,*R*)- and (*S*,*R*)-1,4-fluoro allylic alcohols **48** in a stereodivergent manner (Scheme 19c). The high stereoselectivity observed in both transformations has been explained through the involvement of NHC-catalyzed vinylogous ring opening of the chiral epoxy enal, generating the key azolium dienolate intermediate (Scheme 20).

The latter, in turn, undergoes amination or fluorination through conformations **A** or **B**, respectively. The chiral portion of the catalyst sterically hampers the approach of the reagent on the *Re* face of the dienolate, thus leading to the formation of a single diastereoisomer of the product. The simplified transition state structures **TS-1** and **TS-2** allow the experimental observation that inherent substrate chirality does not affect the stereoselectivity to be explained, which is only determined by the NHC catalyst.

Predicting the regioselectivity of the epoxide opening is often not a trivial task. To provide more elements to organic chemists in developing new and broadly applicable methodologies and organocatalysts, Lan and Wei reported a DFT study aimed at rationalizing the origin of regio- and stereocontrol of NHC-carbene-catalyzed ring-opening/fluorination reaction of epoxy enals.^105^ The computational results help to identify the stereo- and regio-determining step, together with the key noncovalent interactions involved in the stereocontrol.

**Scheme 21:**
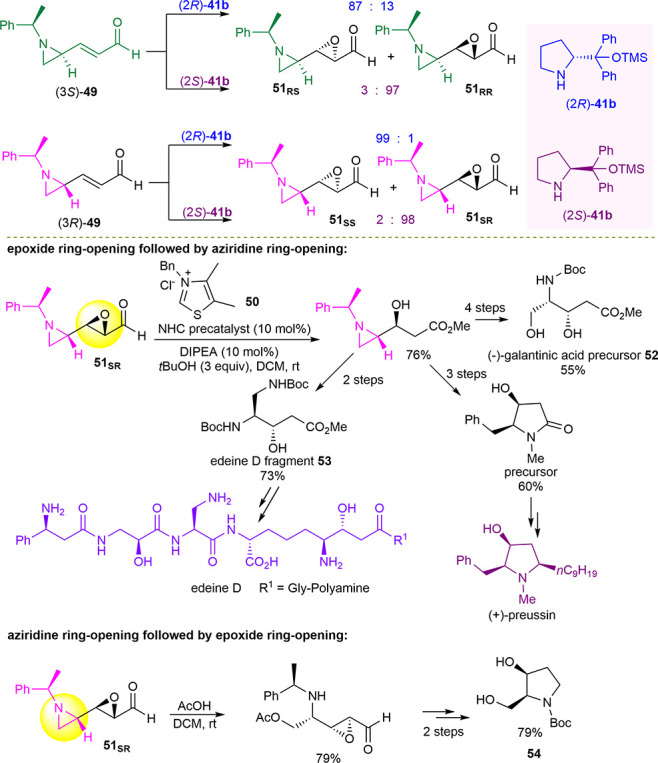
Asymmetric Synthesis and Manipulation of Chiral Synthons Bearing Three Consecutive Functional Groups, Including Aziridine, Epoxide, and Aldehyde

The same epoxidation system was applied by Ha and Yang to synthesize a novel synthon, 3-(aziridine-2-yl)oxirane-2-carbaldehyde **51**, containing the two vicinal chiral epoxide and aziridine moieties, contiguous to an aldehydic group (Scheme 21).^106^ With respect to the simple pyrrolidine, the Jørgensen–Hayashi catalyst **41b** gave better yields (72–75% vs 32%) and higher diastereoselectivity (up to 99:1 vs 66:34) in the epoxidation performed in ethanol with H_2_O_2_. In particular, the “matching” effect between (3*S*)-(aziridine-2-yl)acrylaldehyde **49** and the catalyst (2*S*)-**41b** led to stereoselectivity (97:3 dr) higher than that of the “mismatched” pair (3*S*)-**49** and (2*R*)-**41b** (87:13 dr). On the other hand, both enantiomeric forms of the catalyst gave an excellent diastereomeric ratio starting from (3*R*)-(aziridine-2-yl)acrylaldehyde **49** (98:2–99:1 dr). The synthetic utility of the multifunctional synthon **51** was demonstrated in the regioselective ring opening of the three-membered rings to prepare useful frameworks for drug syntheses. Through a first ring opening of epoxide followed by aziridine ring elaboration, the syntheses of (−)-galantinic amino acid precursor **52**, 3-hydroxy-4,5-diaminopentanoic acid **53**, which is a fragment of antibiotic edeine D, and the formal synthesis of potent antifungal (+)-preussin were accomplished. On the contrary, by reversing the ring-opening sequence, 2-hydroxymethyl-3-pyrrolidine **54**, a key building block to obtain many pharmacologically active compounds, was accessible in three simple steps in 63% overall yield (Scheme 21).

**Scheme 22:**
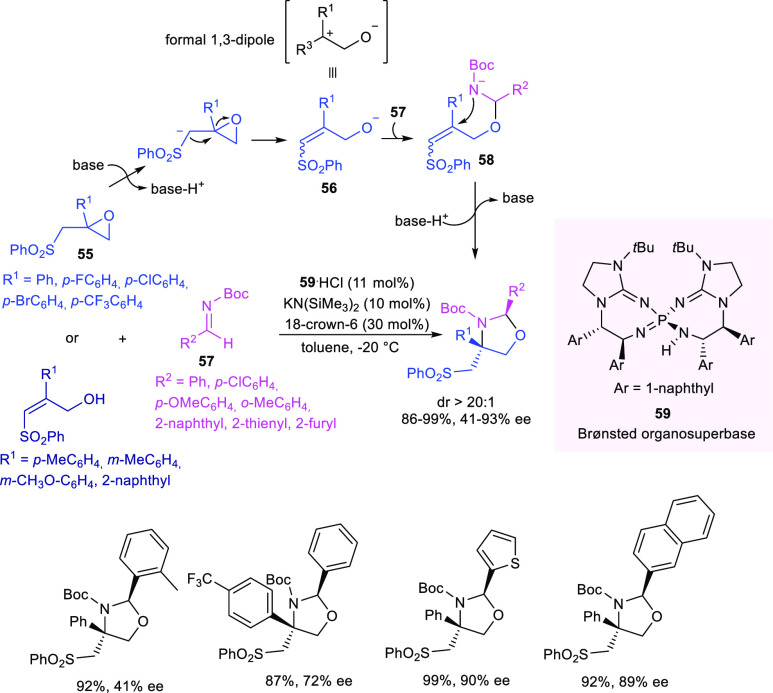
Enantioselective Formal [3 + 2] Cycloaddition of β,γ-Epoxysulfones with Imines Catalyzed by a Chiral Organosuperbase

In the same year, Terada reported the formal [3 + 2] cycloaddition of β,γ-epoxysulfones with imines for the diastereo- and enantioselective synthesis of 1,3-oxazolidines, having a tertiary and a quaternary stereocenter, catalyzed by a chiral organosuperbase bis(guanidino)iminophosphorane **59** (Scheme 22).^107^ The Brønsted base deprotonated β,γ-epoxysulfones **55** at the α-position of the sulfonyl group, generating the alkoxide intermediate **56**, which behaved like a synthetic equivalent of a 1,3-dipole. The organocatalyst **59** controlled the enantioselectivity in the addition to imine **57**, forming intermediate **58**, which underwent the diastereoselective ring closure to 1,3-oxazolidine via intramolecular aza-Michael addition. The strong basicity of the catalyst is crucial for promoting the intramolecular aza-Michael addition of the anionic intermediate **58** on the poorly electrophilic β,β-disubstituted sulfone. Moreover, since the active organocatalyst was in situ generated by treating **59**·HCl with KN(SiMe_3_)_2_, the addition of 30 mol % of 18-crown-6 was found to be necessary to improve the nucleophilicity of the anion **58**. Enantioenriched 1,3-oxazolidines were obtained in high yields and enantioselectivity (72–93% ee) independently by the substitution pattern in the β-aromatic moiety of the epoxide, whereas β-methyl-substituted sulfonyl epoxides did not afford the desired product. 2-Naphthyl-substituted and heteroaryl imines were well-tolerated as well as aryl imines, bearing both electron-withdrawing and electron-donating groups (72–93% ee), except in the case of *ortho*-tolyl substitution (41% ee).

By carrying out some control experiments, the authors demonstrated that the diastereocontrol in the intramolecular aza-Michael step was determined to a greater extent by substrate control, with the assistance of the chiral catalyst. In addition, it was demonstrated that (*Z*)-**58** was much more reactive than (*E*)-**58** under the usual conditions. Furthermore, the enantiocontrol in the addition of the *Z*-isomer of alkoxide **56** to the imine was significantly higher compared to that observed in the addition of *E*-isomer (82% ee vs 46% ee), thus suggesting the involvement of the (*Z*)-alkoxide **56** in the epoxide ring-opening step.

**Scheme 23:**
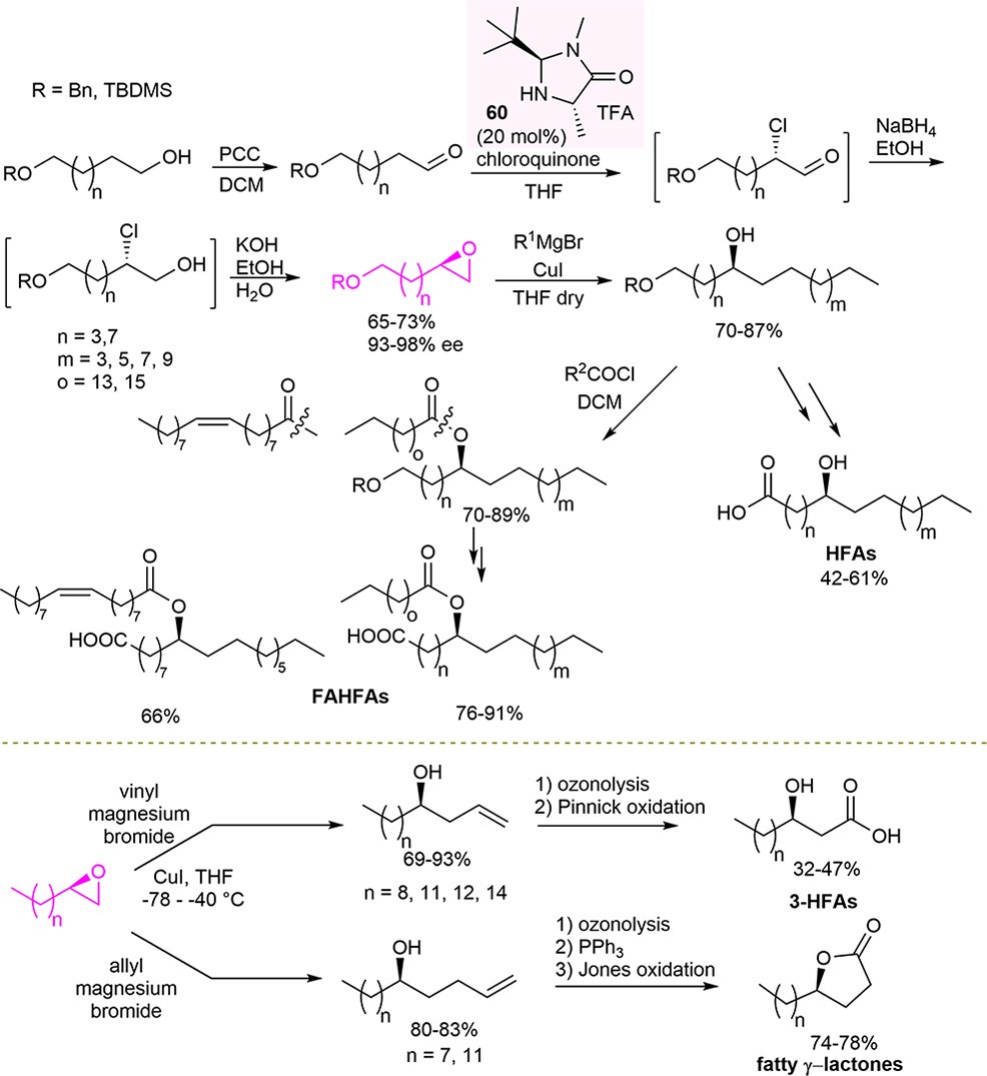
Synthesis of Enantioenriched Hydroxy Fatty Acids, Fatty Acid Esters of Hydroxy Fatty Acids, and Fatty γ-Lactones via Asymmetric Organocatalytic Synthesis of Terminal Epoxides

In 2019, Kokotos developed a general methodology for the asymmetric synthesis of hydroxy fatty acids (HFAs), fatty acid esters of hydroxy fatty acids (FAHFAs), and fatty γ-lactones via ring opening of enantioenriched terminal epoxides by a Grignard reagent (Scheme 23).^108,109^ Hydroxy fatty acids have been found to interact more effectively with free fatty acid receptors than the corresponding nonhydroxylated analogues. These bioactive molecules play important roles, also as signaling molecules, in numerous physiological and inflammatory processes. As an example, FAHFAs are involved in controlling and modulating several cellular activities with potential anti-inflammatory and antidiabetic effects, such as the well-known palmitic acid esters of hydroxy stearic acids. Terminal epoxides were obtained through a one-pot procedure, involving enamine-based enantioselective chlorination of aliphatic aldehydes, catalyzed by MacMillan’s third generation imidazolidinone organocatalyst **60**, followed by reduction and basic treatment. By simple synthetic transformations of enantioenriched intermediates, a library of FAHFAs has been obtained, bearing the hydroxy group at different positions of the chain. The substitution motifs were built by choosing the appropriate starting aldehyde, in turn, obtained from readily available monoprotected α,ω-diols, and the proper alkyl magnesium bromide. Subsequently, the hydroxy group was acylated with different fatty acyl chlorides, followed by alcohol deprotection and Jones oxidation to give different enantioenriched saturated and unsaturated FAHFAs in high enantiomeric purity (up to 93% ee). In addition, by a slightly modified strategy, enantioenriched 3-hydroxy fatty acids and fatty γ-lactones were easily prepared via epoxide ring opening with vinylmagnesium bromide or allylmagnesium bromide, respectively, followed by ozonolysis and subsequent oxidation (Scheme 23). Finally, considering the utility of deuterated FAHFAs and HFA as internal standards in biological and mass spectrometry studies, the development of a practical protocol for the synthesis of deuterated analogues enriched the study.

**Scheme 24:**
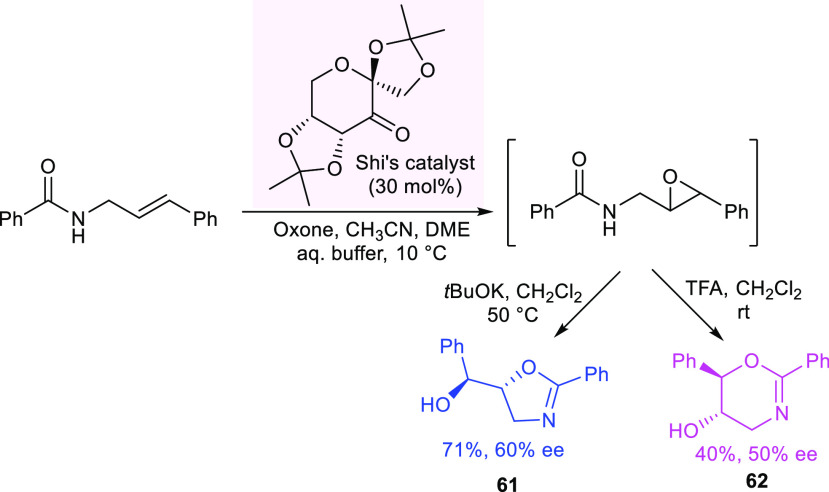
Asymmetric Synthesis of Oxazolines and Dihydrooxazines through an Epoxidation/Regioselective Cyclization Sequence

In 2018, Kokotos developed an organocatalytic methodology for the selective synthesis of racemic oxazolines and dihydrooxazines starting from allyl amides.^110^ The regioselectivity of the cyclization, in general, followed Baldwin’s empirical rules and depended on the substitution pattern of the substrate. Using simple and complementary reaction conditions, the epoxidation/cyclization sequence allowed the inherent selectivity of cyclization to be overcome, affording either the five-membered or the six-membered rings. The authors reported an unoptimized asymmetric variant using 30 mol % of Shi’s catalyst and oxone as the terminal oxidant, obtaining oxazoline **61** in 71% yield and 60% ee and dihydrooxazine **62** in 40% yield and 50% ee (Scheme 24).

**Scheme 25:**
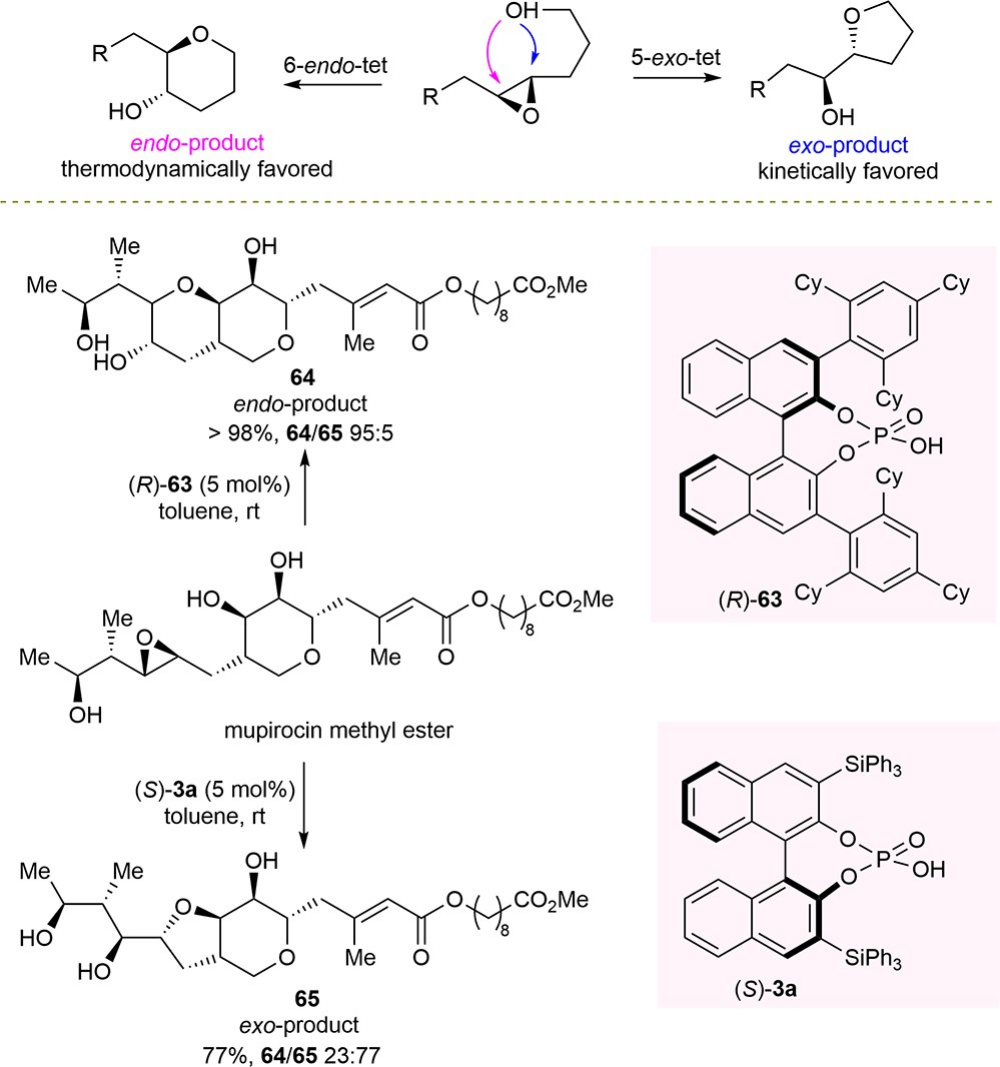
Regiodivergent Chiral Phosphoric-Acid-Catalyzed Intramolecular Ring-Opening Reaction of Antibiotic Mupirocin Methyl Ester

Recently, Zimmerman and Nagorny described the first example of a catalytic strategy which enabled the control of the regioselectivity in the intramolecular epoxide ring opening of epoxyalcohols to generate *exo*- and *endo*-products, tetrahydrofurans and tetrahydropyrans, respectively.^111^ Using chiral phosphoric acids, the regiodivergent cycloisomerization of epoxide-containing antibiotic mupirocin methyl ester into either five- and six-membered cyclic ether derivatives has been accomplished (Scheme 25).

Under standard conditions, in the presence of achiral acidic and basic catalysts or reagents, no regiocontrol was observed. On the contrary, the BINOL-derived catalyst (*R*)-**63** was found to significantly affect the regioselectivity of the cycloisomerization, which, in contrast to that predicted by Baldwin’s rules, favored the *endo*-product **64**. On the other hand, *S*-configured BINOL-derived catalyst **3a** turned out to be the most effective to achieve higher *exo*-selectivity, although with moderate regiocontrol compared to the *endo*-selectivity case. The authors investigated the mechanism both experimentally and by DFT calculations. Zimmerman’s state-of-the-art quantum chemical solutions gave the description of the potential energy surface fully coherent with the experimental findings. A concerted and highly synchronous mechanism was proposed for the reaction, and detailed reaction pathways were depicted for the formation of *exo*- and *endo*-products. Consequently, the origin of the regiocontrol was ascribed to steric hindrance into key transition states between epoxide alkyl substituents and the substituents of the BINOL-derived phosphoric acid.

**Scheme 26:**
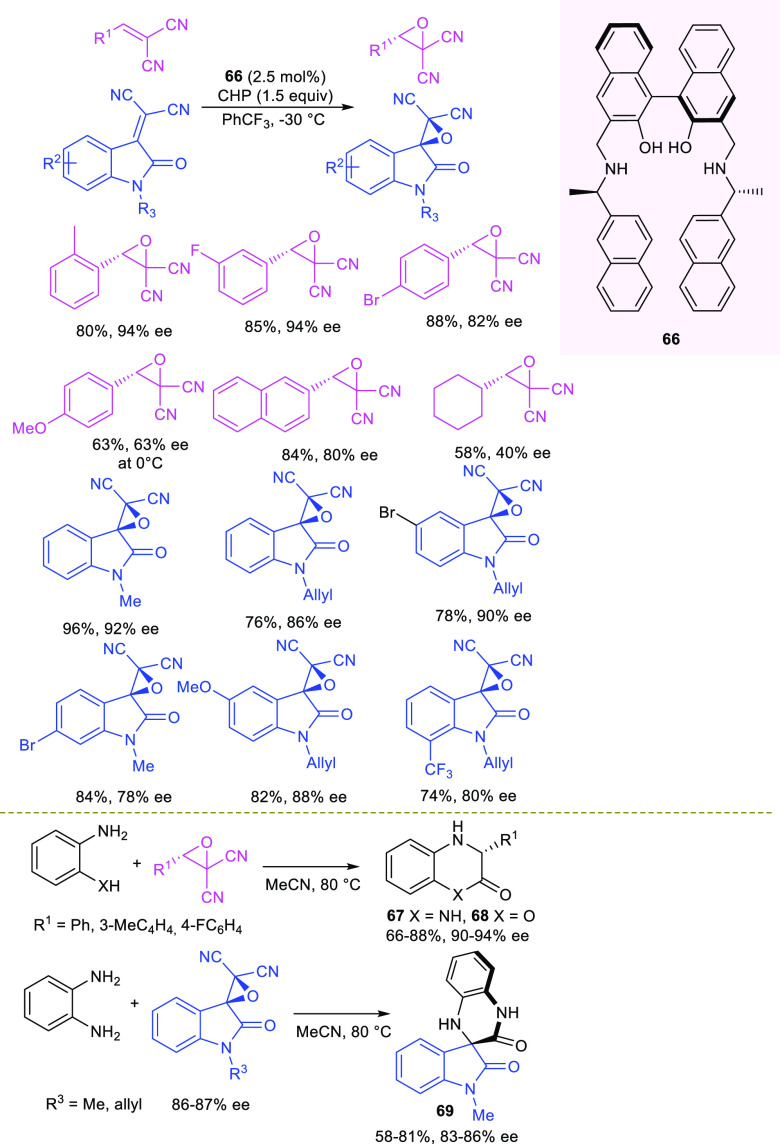
Asymmetric Synthesis of Dihydroquinoxalinones and Dihydroquinoxalinyl Spiro-Oxindole via Enantioenriched *gem*-Dicyanoepoxides

In 2021, Arai and co-authors reported the synthesis of enantioenriched heterocycles via a stepwise asymmetric epoxidation of alkylidenemalononitriles with cumyl hydroperoxide (CHP) organocatalyzed by a chiral *C*_2_-symmetric aminomethylbinaphthol **66** followed by opening reaction of *gem*-dicyanoepoxides (Scheme 26).^112^ A previously reported one-pot enantioselective organocatalytic epoxidation of alkylidenemalononitriles to *gem*-dicyanoepoxides, followed by ring opening, demonstrated useful access to enantioenriched 3-aryl piperazin-2-ones.^113^ The oxidative system illustrated in Scheme 26 turned out to be effective for the enantioselective epoxidation of simple alkylidenemalonitriles as well as more congested isatilidenemalonitriles, achieving good to high enantioselectivity (63–96% ee). The chemical behavior of *gem*-dicyanoepoxides to act as synthetic equivalents of dication ketene^113−115^ enabled their transformation into dihydroquinoxalinones **67**, 1,4-benzoxazin-2-one **68**, and dihydroquinoxalinyl spirooxindole **69**, while maintaining the enantiomeric excess, when reacting with 1,2-diamines or 2-aminophenol. The mechanism would involve a regioselective S_N_2 ring-opening reaction of the isolated enantioenriched *gem*-dicyanoepoxides by the binucleophilic reagent, followed by an intramolecular amidation of the acylcyano intermediate. A reaction model was proposed to explain the observed stereochemical outcome, where the alkylidenemalononitrile acts as a bidentate substrate with both nitrile groups H-bonded by phenolic groups in the *C*_2_-symmetric catalytic pocket, exposing the *Re* face to the attack of pronucleophile CHP, in turn, activated by the basic secondary amine moiety.

**Scheme 27:**
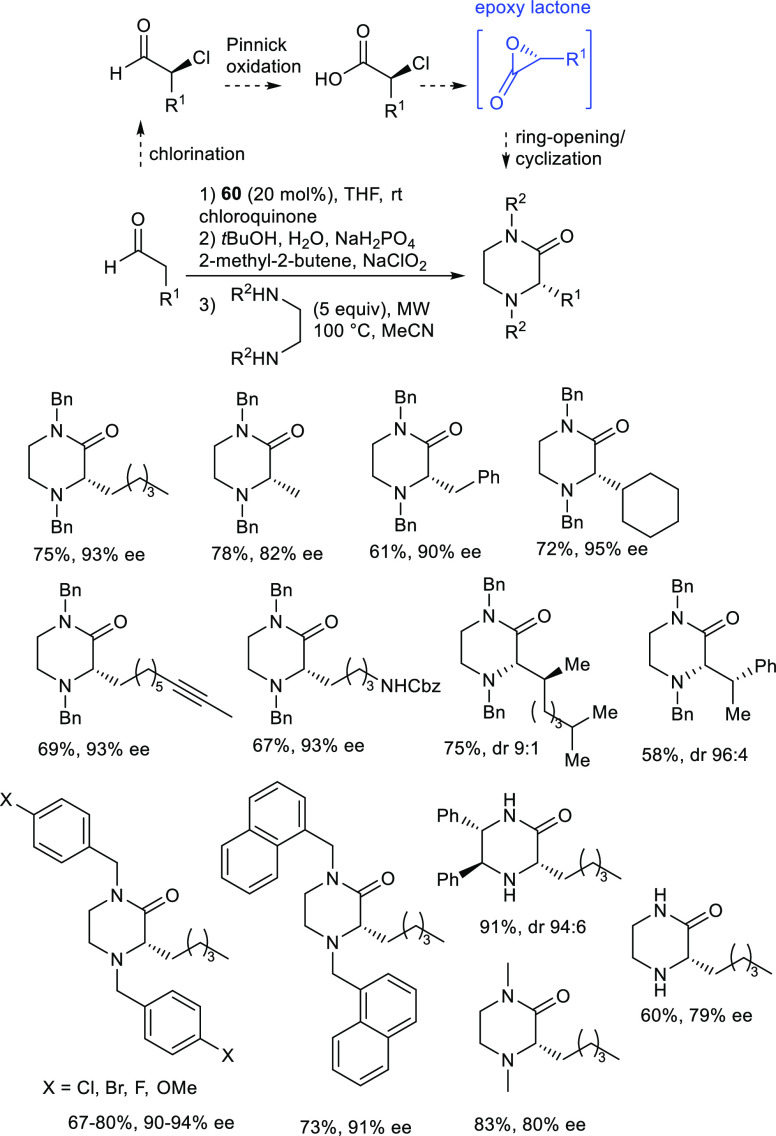
Enantioselective Synthesis of 2-Oxopiperazines from Aldehydes via Transient Epoxy Lactone Intermediate

In 2016, a transient and short-lived epoxy lactone intermediate was detected for the first time by Kokotos and co-authors, as a key intermediate in a four-step reaction sequence for the enantioselective synthesis of 2-oxopiperazines, starting from readily available aldehydes (Scheme 27).^116^ The reaction sequence involves enantioselective chlorination, oxidation, nucleophilic substitution, and cyclization in just one operation. The enantioselective chlorination was performed using a modified protocol promoted by MacMillan’s catalyst **60**,^117^ via enamine catalysis and chloroquinone as the electrophilic agent.

The following Pinnick oxidation afforded α-chloroheptanoic acid, which, upon treatment with diamine at 100 °C under microwave (MW) irradiation, was converted into highly enantioenriched 2-oxopiperazines. The authors demonstrated that the nucleophilic substitution of chlorine by the nitrogen of the diamine occurred with retention of stereochemistry. Therefore, the direct S_N_2 pathway, which would be competitive at room temperature, was not operative at 100 °C. On the contrary, the close carboxylic group displaced the chloride, generating a labile epoxy lactone intermediate, which was, in turn, opened by the diamine and, after cyclization, led to the enantioenriched heterocycle. The involvement of an epoxy lactone intermediate was confirmed by HRMS studies.

**Scheme 28:**
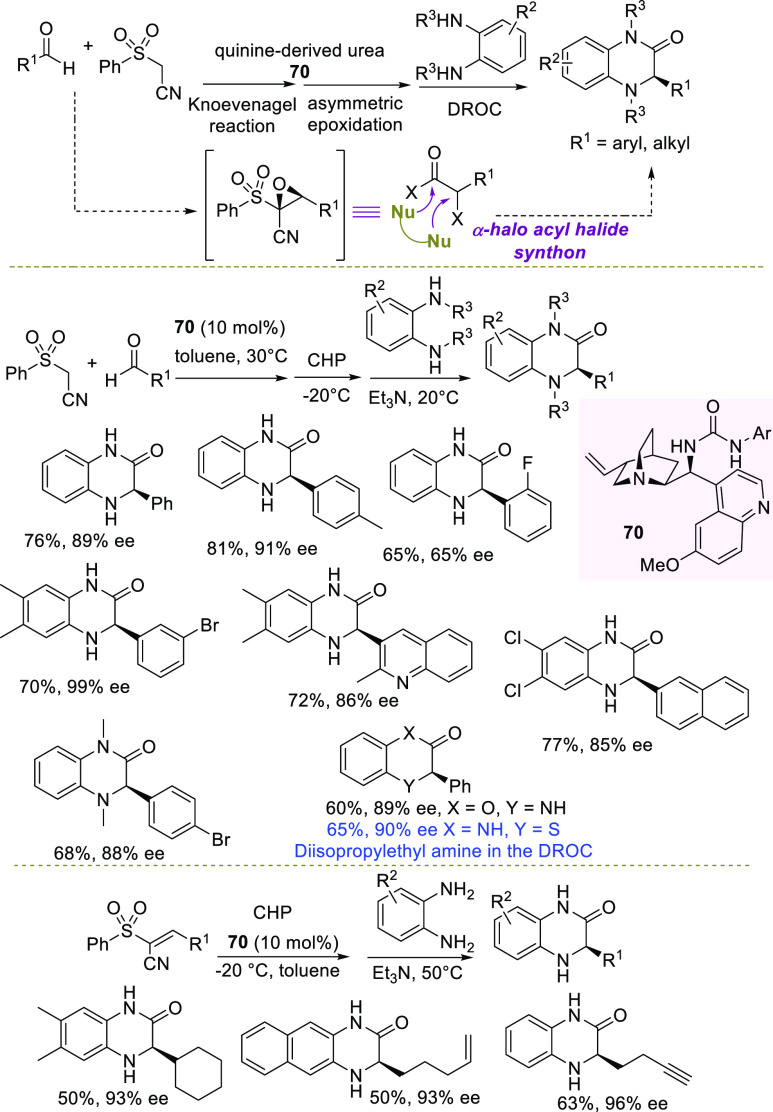
Enantioselective One-Pot Catalytic Strategy to Dihydroquinoxalinones via Novel 1-Phenylsulfonyl-1-cyano-Enantioenriched Epoxides Catalyzed by Quinine-Derived Urea

Considering the importance of dihydroquinoxalinones as privileged scaffolds in medicinal chemistry, Lattanzi et al. recently developed a general enantioselective methodology to obtain these heterocycles, overcoming some of the drawbacks of previously reported methods, which required multistep preparation of the reagents and often suffered from a limited substrate scope. The new strategy is based on the use a new class of epoxides, i.e., phenylsulfonyl cyanoepoxides, masking α-halogenated acyl halides, able to react with *ortho*-phenylenediamines to give the desired heterocycles (Scheme 28).^118^

More ambitiously, the access to heterocycles envisioned a one-pot protocol starting directly from commercially available aldehydes, (phenylsulfonyl)acetonitrile, and using a simple and recyclable organocatalyst. To this end, a sequence consisting of a Knoevenagel reaction/enantioselective epoxidation, both promoted by quinine derived urea **70**, was followed by a domino ring-opening cyclization (DROC) in the presence of a *ortho*-phenylenediamine and an acid scavenger. Key to the success of the entire strategy was the presence of the sulfonyl group, which served as a strong hydrogen-bonding acceptor, able to direct the stereocontrol in the formation of the epoxide. After a first optimization study, the methodology was applied to a variety of aromatic and heteroaromatic aldehydes and substituted *ortho*-phenylenediamines, as well as bulkier *N*-methyl *ortho*-phenylendiamines, affording directly the nitrogen-protected heterocycles. The products were obtained in good to high yields and high to excellent enantioselectivity. The procedure was successfully applied in the synthesis of the corresponding model oxygen and sulfur-based heterocycles, achieving comparable results. Noteworthy, this is the first methodology leading to benzothiazinones in high enantiomeric excess.

Moreover, heterocycles bearing an alkyl moiety have been prepared, starting from the corresponding alkenes. Double and triple bonds were also introduced in the side chain of the heterocycle, as chemical groups useful for postfunctionalizations. Finally, the catalyst could be recycled and reused for at least four runs without any loss of efficiency. DFT calculations helped to elucidate the origin of the enantiocontrol in the epoxidation step, essentially based on the crucial role played in the transition state by the sulfonyl group, in establishing an effective network of hydrogen-bonding interactions with the bifunctional organocatalyst. The latter also provided assistance through the basic tertiary nitrogen, first in deprotonation of the oxidant and then in the leaving group departure.

## 6 Conclusion and Outlook

In the past few years, we have assisted with significant developments of ARO reactions of epoxides, operating under organocatalytic conditions. The new achievements, herein illustrated, indicate the role played by epoxides as first-class compounds in organic synthesis to access functionalized intermediates and heterocycles. As expected, in addition to bifunctional organocatalysts, well-represented in ring-opening reactions provided by the families of chiral phosphoric acids, other chiral acids have started to become useful. Arguably, proton activation will be increased in the years to come, expecting the development of new organocatalysts. Interestingly, simple H-bonding donors, although being less effective, began to emerge, suggesting that more efforts should be directed to expand ARO reactions, including this class of promoters. In line with the current need to develop evermore green and convenient reactions, asymmetric organocatalytic one-pot processes, combining synthesis and ring-opening reaction of the epoxides, should be designed. To face this more challenging issue, organic chemists could benefit from different activation strategies and organocatalysts. Moreover, the combination with metal and photocatalyses might help to improve the still major limitation observed in the ring-opening step, such as the involvement of carbon nucleophiles with respect to heteroatom-based reagents.
